# Genetic diversity and structure of Slovenian native germplasm of plum species (*P. domestica* L., *P. cerasifera* Ehrh. and *P. spinosa* L.)

**DOI:** 10.3389/fpls.2023.1150459

**Published:** 2023-03-21

**Authors:** T. Ternjak, T. Barreneche, M. Šiško, A. Ivančič, A. Šušek, J. Quero-García

**Affiliations:** ^1^ Faculty of Agriculture and Life Sciences, University of Maribor, Hoče, Slovenia; ^2^ INRAE, Univ. Bordeaux, UMR BFP, Villenave d’Ornon, France

**Keywords:** *Prunus* spp., plum, genetic resources, genetic diversity, genetic structure, cpDNA, SSR

## Abstract

Slovenia has particular climatic, soil, geographic and historical conditions that lead to long tradition of plum cultivation and use. In this work, a set of 11 SSR and three universal cpDNA markers, as well as flow cytometry, were used to (1) evaluate the genetic diversity of 124 accessions of the three *Prunus* species (*P. domestica* L., *P. cerasifera* Ehrh., and *P. spinosa* L.), (2) investigate the possible involvement of *P. cerasifera* and *P. spinosa* species in *P. domestica* origin, (3) study the genetic relationships and variability among the most typical *P. domestica* accessions present in Slovenia. Ten haplotypes of cpDNA were identified and clustered into three groups according to the Neighbor-Joining analysis (NJ). All 11 SSR primer pairs were polymorphic, revealing 116 unique genotypes. A total of 328 alleles were detected with an average value of 29.82 alleles per locus, showing relatively high diversity. Bayesian analysis of genetic structure was used to identify two ancestral populations in the analyses of all three species as well as in a separate set consisting of *P. domestica* material only. Principal Coordinate Analysis (PCoA) showed that accessions clustered largely in agreement with Bayesian analysis. Neighbor-Joining analysis grouped 71 P*. domestica* accessions into three clusters with many subgroups that exhibited complex arrangement. Most accessions clustered in agreement with traditional pomological groups, such as common prunes, mirabelle plums and greengages. In this study, the analyses revealed within *P. domestica* pool valuable local landraces, such as traditional prunes or bluish plums, which seem to be highly interesting from a genetic point of view. Moreover, complementary approaches allowed us to distinguish between the three species and to gain insights into the origin of plum. The results will be instrumental in understanding the diversity of Slovenian plum germplasm, improving the conservation process, recovering local genotypes and enriching existing collections of plant genetic resources.

## 1 Introduction

Plums are considered as one of the most colourful and delicious temperate fruits, with a wide range of fruit sizes and shapes (**Figure S1A**), and are well adapted to different growing conditions (Milošević and Milošević, 2018). They are deciduous fruit trees belonging to the stone fruits classified in the Rosaceae family and genus *Prunus*. It is thought that the main existing European plum species, *P. domestica* L. (European plum) (**Figure S1B**), *P. cerasifera* Ehrh. (Cherry plum, myrobolan) (**Figure S1C**) and *P. spinosa* L. (blackthorn, sloe) (**Figure S1D**) descended from an ancestor that migrated from East Asia (Chin et al., 2014).

According to Insero et al. (1991), Eurasian plum is one of the most important and globally distributed *Prunus* group, which includes the hexaploid European plum, *P. domestica* (2n = 6x = 48), and *P. insititia*, which was considered a distinct species by some authors in the past and is now classified as a subspecies of *Prunus domestica* (*P. domestica* subsp. *insititia* C. K. Schneider). European plum cultivars have been generally classified into different pomological groups based on morphological characteristics as well as fruit use, including large-fruited European plums, prunes, egg plums, greengages, mirabelles, damsons, bullaces and St. Julien plums (Zhebentyayeva et al., 2019). Prunes are mainly processed for drying, but can also be used fresh, for distilling, baking and jams. They differ from the other plum groups, as the whole fruit can be dried, without fermenting, due to their high sugar content (Faust and Suranyi, 1999). Traditionally, egg plums produce yellow fruits and are mainly used for canning, while greengages usually produce round, small, green fruits that are considered the sweetest among plums (Topp et al., 2012). Damsons, bullaces, mirabelles and St. Julien plums are mostly used for food processing. St. Julien plums are also commonly used as rootstocks. Very sweet and highly aromatic mirabelle plums have yellow to orange fruits, while damsons and bullaces form yellow or greenish to bluish, small, round to oval fruits (Gaši et al., 2020).

*P. cerasifera* (2n = 2x = 16), the diploid cherry plum, contains wild and cultivated genotypes used as ornamentals, for their fruits, or as rootstocks. Another important species closely related to the cultivated plum is the tetraploid *P. spinosa* (2n = 4x = 32). Some authors consider this wild species as one of the possible ancestors of *P. domestica*. There are still numerous questions about the genetic origin of *P. domestica*. Most authors support the theory that hexaploid *P. domestica* is an interspecific hybrid between diploid *P. cerasifera* and tetraploid *P. spinosa* (Rybin, 1936; Crane and Lawrence, 1938; Werneck, 1961; Decroocq et al., 2004; Bortiri et al., 2009; Ilgin et al., 2014). In contrast, other authors discuss that European plum could have been a result of alloploidization of polyploid forms of *P. cerasifera* (Bajashvili, 1990; Zohary, 1992; Decroocq et al., 2004; Okie and Hancock, 2008; Depypere et al., 2009; Weiss et al., 2012) or *P. spinosa* (Bajashvili, 1990; Woldring, 2003; Liu et al., 2007). Hedrick (1911) hypothesised that *P. domestica* was derived from *P. spinosa*, with *P. insititia* being an intermediate form between the two. More recent results support the possibility that *P. domestica* arose from interspecific hybrids with a hexaploid chromosome complex of *P. cerasifera*, *P. spinosa*, and potential progenitor species from the Eurasian plum cluster (Zhebentyayeva et al., 2019).

Slovenia, part of the former Republic of Yugoslavia, located in the northwest of the Balkan Peninsula, has a long history of cultivating plum species. It is believed that the Balkan Peninsula is one of the richest regions in Europe in terms of plum fruits, with highly valuable genetic material (Vukojevic et al., 2012). Moreover, according to Vavilov (1949), it belongs to one of the eight independent centres of origin of the most important cultivated plants in the world: the Mediterranean Centre. Mihai et al. (2012) considered *P. domestica* as an indigenous species in the Balkans, represented by thousands of local biotypes that have been cultivated in this area for centuries and are part of the local culture.

On the territory of present-day Slovenia, the first systematic plum cultivation took place during the Roman period (Kazija et al., 2014). In the Middle Ages, plums were grown mainly in village gardens, monasteries and small orchards (Rencelj and Pozrl, 2015). In the nineteenth century, plum was the most prevalent fruit tree species, with the leading *P. domestica* landrace, the common prune (‘Domača češplja’, syn. ‘Bistrica’, ‘Požegača’, ‘Dolanka’), also known in the wider area of the Balkan Peninsula. Later, other varieties of European plum spread, and in the last two decades the selection was also enriched by some varieties of Chinese-Japanese origin (Štampar et al., 2005).

Different plum varieties were used both, for fresh consumption and for processing. In the eastern (Styria and Lower Carniola) and western (Goriška brda, Soča and Vipava valleys) parts of the country, plum drying was seasonal, but a very important source of income for rural population, especially women from small farm households (Pockar, 1982). The tradition of peeling and drying plums, called ‘prunes’ (‘prunele’), was brought to Slovenia by French soldiers that deserted Napoleon’s army at the beginning of the 19^th^ century (Pockar, 1982). The prevailing genotype used for plums drying was landrace ‘Domača češplja’. Distillation of plum brandy (slivovec, slivovica) was also an important source of income for rural folks and usually made with common prune ‘Domača češplja’ (Rencelj and Pozrl, 2015).

In Styria (NE Slovenia), the sweet and tasty fruits of another important landrace were used. The so-called ‘plavkica’ (directly translated - small bluish fruits) is an early flowering, round, small fruited, dark blue or violet-blue plum, with greenish to yellowish colour of the flesh that fully or partly adheres to the stone. Written information about this landrace is very scarce. Mišić (1979) stated that ‘plavkica’ (from this point referred as Bluish plum) was one of the widespread varieties grown in Slovenia, while Honzak (1968) wrote about seedlings of cultivated local varieties used as a rootstocks.

Highly specific climatic, soil, geographic and historical conditions of Slovenia resulted in valuable plum diversity and many unique genotypes could harbour important characteristics for subsequent breeding and food security. Unfortunately, the use of local plum varieties and the number of varieties in cultivation have declined significantly in the recent decades (Usenik et al., 2007). Environmental changes, land use and intensified cultivation practises, introduction of new cultivars, as well as economic and social changes, have led to significant erosion of indigenous genetic diversity (Benediková and Giovannini, 2011; Luthar, 2018). In addition, *Plum pox virus* disease (Sharka) has greatly affected plum cultivation as well as the distribution of the species in its natural habitat, resulting in plum germplasm depletion (Usenik et al., 2007; Kazija et al., 2014). Climate change is one of the biggest challenges facing food production. Therefore, more sustainable agricultural practises need to be adopted, which go hand in hand with breeding strategies. In order to create new cultivars, well adapted to these challenges, exploring a broader genetic base, including primitive indigenous germplasm, is essential to the success of current breeding programmes (Campoy et al., 2016).

To develop more sustainable solutions for the future it has never been more important, to systematically collect, record, evaluate, and conserve the most valuable genetic material (Vukojevic et al., 2012). In the past, studies on the genetic diversity of *P. domestica* were quite scarce due to the polyploid nature of the species, but recently plum germplasms from different countries have been evaluated (Kazija et al., 2014; Sehic et al., 2015; Urbanovich et al., 2017; Urrestarazu et al., 2018; Manco et al., 2019; Zhebentyayeva et al., 2019). Genotyping of autochthonous genetic resources allows identification of synonyms and homonyms, determination of parentage/origin, and identification/evaluation of important genotypes (Gaši et al., 2020). Additional studies, based on agro-morphological and molecular data are needed to assess the current status of diversity and genetic potential of resources and to prepare effective conservation management as well as increase their use for research, exchange, breeding and cultivation (Kaufmane et al., 2002; Blazek, 2007).

The present research was set up to investigate the status of traditional plum germplasm as well as the genetic relationships and variability among accessions belonging to three *Prunus* species (*P. domestica*, *P. cerasifera* and *P. spinosa*) representing the Slovenian plum gene pool. Based on historical and economic importance, the most frequently present traditional plum genotypes in Slovenia probably belong to the species *P. domestica*. Therefore, our work focused on this species with particular interest on Bluish plum and common prune. In the present study, we used a set of SSR and cpDNA markers to: 1) evaluate the relationship among the three *Prunus* species (*P. domestica*, *P. cerasifera* and *P. spinosa*) 2) examine the possible involvement of *P. cerasifera* and *P. spinosa* species in the origin of *P. domestica* 3) investigate the genetic relationships and structure among *P. domestica* accessions collected *in situ* in the Slovenian landscape.

## 2 Materials and methods

### 2.1 Plant material

The study included 124 accessions (**Table S1**). Among these, 54 accessions were collected *in situ*: in the wild, along roads, in home gardens, on abandoned estates and in orchards. The material was collected mostly throughout Slovenia with the emphasis on the north-eastern part, which is considered one of the typical plum-growing regions in Slovenia (Pockar, 1982), while two samples were found in Styria region of Austria and one accession was brought from Croatia. In addition, 70 samples were obtained from various *ex situ* collections: 38 from the Slovene Plant Gene Bank (SPGB) of the Faculty of Agriculture and Life Sciences (Maribor), 21 from the Plum French National Collection (PFNC) of the ‘Centre de Ressources Biologiques - CRB *Prunus Juglans*’, French National Research Institute for Agriculture, Food and Environment, (INRAE) (Nouvelle Aquitaine Bordeaux), and eleven from different botanical gardens or private collections from Slovenia (Maribor and Ljubljana) and Austria (Vienna and Graz). Of the 124 studied accessions, a set of 14 internationally known cultivars was used as reference. The accessions from the PFNC Collection were added in order to compare the genetic resources of Slovenian plum with a sample representing a large diversity within the species. This collection has been well described morphologically in INRAE multispecies integrative information system dedicated to plant and fungi pests (GnpIS, https://urgi.versailles.inra.fr/gnpis), as well as studied with molecular markers by different authors (Horvath et al., 2011; Urrestarazu et al., 2018; Zhebentyayeva et al., 2019). Prior to analysis, all accessions were classified into three different species groups (*P. domestica*, *P. cerasifera* or *P. spinosa*) based on data from the existing collections and our morphological descriptions.

The studied material was also classified according to its status, e.g., wild genotype, landrace, or improved variety. Based on passport data from the existing *ex situ* collections and according to Campoy et al. (2016) and the bibliography, improved varieties were considered “early selections” (such as ‘President’) or modern breeding cultivars (such as ‘Topper’), depending on the period when they were selected.

### 2.2 DNA extraction

Total genomic DNA was isolated following the CTAB protocol described by Doyle and Doyle (1987) with some modifications: DNA was precipitated by adding 60 μl sodium acetate, and samples were incubated at –20°C for 20 min without addition of resuspension buffer or RNAse. DNA concentration was quantified using a fluorimeter (Hoefer DQ 300, Holliston, USA), and quality was checked by electrophoresis on a 3% agarose gel. Products were visualised under UV light (302 nm), using a Benchtop UVP UV Transilluminator (Analytik Jena, Jena, Germany).

### 2.3 Assessment of ploidy level

Relative DNA content was estimated by flow cytometry using DAPI (4’,6’-diamidino-2-phenylindole) staining in Otto buffers. Samples were prepared according to Bohanec (2003) using *Trifolium repens* L. as an internal standard with a 2C nuclear DNA content of 2.07 pg (Arumuganathan and Earle, 1991). Ploidy analysis was performed with a CyFlow^®^ Space Flow Cytometer (Sysmex Partec, Goerlitz, Germany) using a linear scale. Histograms were analysed using Flomax software 2.0 (Sysmex Partec, Goerlitz, Germany). The absolute amount of DNA in a sample was calculated according to the protocols proposed by Dolezel (1991), Marie and Brown (1993) and Dolezel and Bartos (2005).

The ploidy level was already determined for the accessions from the PFNC Collection (INRAE, Horvath, unpublished data), accordingly some of the material was used as reference [e.g.: 170 ‘D’Ente double’ (2n = 6x), 181 blackthorn (2n = 4x), 187 myrobolan ‘Agdzadzor’ (2n = 2x)]. For each reference accession, the absolute amount of DNA was calculated and the values were compared between all accessions.

### 2.4 PCR-RFLP analyses

Three universal primers were used for chloroplast DNA (cpDNA) analyses [i.e. HK, K1K2 (Demesure et al., 1995) and VL (Dumolin-Lapegue et al., 1997)]. These primers were previously used in *Prunus* sp. (Bouhadida et al., 2007; Horvath et al., 2008; Horvath et al., 2011).

Amplified fragments were digested with three primer pair–restriction enzyme combinations for K1K2 (*Hinf I*, *Taq I*, *Alu I*) and with two for VL and HK (*Hinf I*, *Taq I*). Analyses were performed by BioGEVES platform (GEVES, Beaucouzé, France) following the protocol described by Horvath et al. (2011). The size of each fragment was evaluated and scored as binary data (presence/absence). Bands were coded as unique combinations of letters (A, B, C, D) and assigned to a specific haplotype. Restriction fragment data were transformed into a binary matrix and the Unweighted Neighbor-Joining dendrogram was created using DARwin 6.0.21 software (Perrier and Jacquemoud-Collet, 2006).

### 2.5 SSR analyses

All individuals were analysed with a set of 11 SSR primer pairs (**Table 1**) developed on different *Prunus* species (peach, sweet cherry and Japanese plum) (Cipriani et al., 1999; Wünsch, 2009; Mnejja et al., 2010). In addition to their position on the *Prunus* reference map, markers were also selected based on their amplification quality and polymorphism, as reported in previously published works. Primers were labelled with Cy5 or Cy5.5 dye according to Sigma-Aldrich (Saint Louis, USA). Polymerase chain reactions (PCR) were performed using HotStarTaq^®^Plus Master Mix Kit (Qiagen, Hilden, Germany), and the annealing temperature was adjusted accordingly for each marker (approximately 5°C bellow the Tm). Amplifications were performed in a Biometra TProfessional thermal cycler (Analytik Jena, Jena, Germany), and the quality of the fluorescently-labelled PCR products was checked. Fragment size analysis was performed using a Beckman CEQ 8000 DNA sequencer (Beckman Coulter Inc., Brea, USA) according to the manufacturer’s instructions. Alleles appear as peaks in CEQ 8000 DNA sequencer and are compared to the standards during scoring to determine their size. Allele fragments were scored for each accession. First, we started with *P. cerasifera* accessions since they are diploids and therefore easier to evaluate. Then, each SSR was characterized by analyzing the peak profiles for all accessions. Following this approach, the accessions of the other two species were evaluated considering the corresponding profile observed in *P. cerasifera*. When *P. cerasifera* accessions had more than two peaks within the theoretical range of allele size expected for a given SSR, we assumed that the corresponding primer pair amplified two loci. We considered this information when scoring the alleles of the polyploid species. Sometimes for the polyploid material the number of peaks did not match the ploidy level, in these cases, allele dosage was based on the height of the peak. Examples of electropherograms with recorded allele fragments are provided in supplementary material (**Figures S2**, **S3**).

**Table 1:** SSR loci, linkage group (LG), repeat motifs and references of the 11 primer pairs used in this study.

Locus	LG	Source species	Primer sequence (5’-3’)	Repeat motif	Reference
UDP96-005	1	*P. persica* (L.) Batsch	For: GTAACGCTCGCTACCACAAA Rev: CCTGCATATCACCACCCAG	(AC)_16_TG(CT)_2_CA(CT)_11_	Cipriani et al. (1999)
BPPCT034	2	*P. persica* (L.) Batsch	For: CTACCTGAAATAAGCAGAGCCAT Rev: CAATGGAGAATGGGGTGC	(GA)_19_	Dirlewanger et al. (2002)
EMPAS12	3	*P. avium* L.	For: TGTGCTAATGCCAAAAATACC Rev: ACATGCATTTCAACCCACTC	(TG)_10_A/GA)_10_AA(GA)_13_	Vughan and Russell (2004)
UCD-CH17	4	*P. avium* L.	For: TGGACTTCACTCATTTCAGAGA Rev: ACTGCAGAGAATTTCCACAACCA	(CT)_11_	Struss et al. (2003)
EMPAS06	4	*P. avium* L.	For: AAGCGGAAAGCACAGGTAG Rev: TTGCTAGCATAGAAAAGAATTGTAG	(CT)_12_	Vughan and Russell (2004)
EMPAS11	5	*P. avium* L.	For: ACCACTTTGAGGAACTTGGG Rev: CTGCCTGGAAGAGCAATAAC	(TC)_25_	Vughan and Russell (2004)
EMPAS14	5	*P. avium* L.	For: TCCGCCATATCACAATCAAC Rev: TTCCACACAAAAACCAATCC	(TC)_10_CCAT(TC)_5_CCAT(TC)_8_	Vughan and Russell (2004)
BPPCT014	5	*P. persica* (L.) Batsch	For: TTGTCTGCCTCTCATCTTAACC Rev: CATCGCAGAGAACTGAGAGC	(AG)_23_	Dirlewanger et al. (2002)
BPPCT025	6	*P. persica* (L.) Batsch	For: TCCTGCGTAGAAGAAGGTAGC Rev: CGACATAAAGTCCAAATGGC	(GA)_29_	Dirlewanger et al. (2002)
CPSCT026	7	*P. salicina* Lindl.	For: TCTCACACGCTTTCGTCAAC Rev: AAAAAGCCAAAAGGGGTTGT	(CT)_16_	Mnejja et al. (2004)
CPPCT006	8	*P. persica* (L.) Batsch	For: AATTAACTCCAACAGCTCCA Rev: ATGGTTGCTTAATTCAATGG	(CT)_16_	Aranzana et al. (2002)

Due to the polyploid nature of the *Prunus* species discussed in this work, calculation of classical genetic diversity parameters is not possible because of the ambiguous allele dosage. Hence, distribution of SSR alleles in the studied *Prunus* species was estimated as follows: number of detected alleles, alleles found in all species, and alleles common only to *P. domestica*, *P. cerasifera* or *P. spinosa.*

Bayesian model-based analysis was applied using the software package STRUCTURE V2.3.4 (Pritchard et al., 2000) to investigate the genetic structure of accessions and estimate the number of subpopulations. This software is suitable for data sets with different ploidy levels. Since the studied individuals were polyploids with ambiguous genotypes, the recessive allele approach was used (Falush et al., 2007). When all species were analysed together (diploids, tetraploids, and hexaploids), genotypes were coded according to Horvath et al. (2011). Among the options offered by the software, we chose the admixture model with correlated allele frequencies (Falush et al., 2007). The parameter K was set between 1 and 20 inferred clusters, with 20 independent iterations for each simulation. For each run, 100.000 burn-in periods followed by 750.000 MCMC (Markov Chain Monte Carlo) replicates, were performed.

The most relevant number of clusters (K) for the analysed data was estimated by calculating ΔK based on the method of Evanno (Evanno et al., 2005) implemented in the Structure Harvester V0.6.94 application (Earl and von Holdt, 2012). The threshold of 90% was chosen to assign a given individual to a population. The raw data (Q-matrix) obtained with the STRUCTURE software were used as a basis for the R package ‘tidyverse’ to visualise the results in the form of bar plots. In a second step, the Structure software was applied to a separate data set of *P. domestica* accessions with the same parameters.

Principal Coordinate Analysis (PCoA) was used to confirm the genetic structure of the three studied species and independently of the *P. domestica* accessions using DARwin 6.0.21 software (Perrier and Jacquemoud-Collet, 2006). This statistical method examines dissimilarities by converting data on distances between individuals into a location in low-dimensional space (e.g., 2D or 3D graphs). The software option single data format was used and allele data for each accession were converted into a binary matrix of presence (1) and absence (0) (**Table S2**). Despite the co-dominant nature of the SSR markers, the results were scored in a dominant manner. As Rouger and Jump (2014) noted, a part of genetic information is lost as a result, nevertheless this method has been successfully used in diversity studies of polyploid *Prunus* species (Horvath et al., 2011; Sehic et al., 2015; Halász et al., 2017; Urrestarazu et al., 2018).

First, dissimilarities (30.000 bootstraps) were calculated with Sokal and Michener index (simple matching):


$$d_{ij}=\frac{u}{m+u}$$

where *d_ij_* represents dissimilarity between units *i* and *j*, *u* is the number of unmatched variables and *m* is the number of matched variables. Information about the presence of the allele is rather confused in polyploids because allele frequencies are not clear. Therefore, the presence and absence modalities must be considered of equal weight, which minimises the loss of information (Noyer et al., 2003). However, some authors (Cordeiro et al., 2003) have used the Jaccard coefficient, which disregards the joint absence of bands in pairwaise comparison and therefore most likely underestimates dissimilarity. On that ground, dissimilarities were also calculated using the Jaccard coefficient and the results were compared. The calculated dissimilarity was transformed into Euclidean distance using the 0.5 power transformation, and the graphical representation of the PCoA results was complemented using the by R package ‘scatterplot3d’. In addition, the calculated dissimilarity was used to estimate the genetic relationships among *P. domestica* accessions. Unweighted Neighbor-Joining (NJ) method was used to generate a tree with DARwin 6.0.21 software (Perrier and Jacquemoud-Collet, 2006). This method has been successfully used in similar studies (Campoy et al., 2016).

## 3 Results

### 3.1 Ploidy level determination of the plum species

According to relative DNA content estimated by flow cytometry, analysis of 124 studied accessions revealed 56 hexaploid, seven tetraploid and 41 diploid accessions (**Table S3**), with 2C nuclear DNA (pg) ranging from 1.35-1.698, 0.85-1.14 and 0.44-0.52, respectively (**Table S4**). The measuring was not successful for six samples, so their ploidy level was considered unknown.

Thirty-three accessions that were classified as *P. domestica* and considered hexaploid prior to analysis, based on recorded data from existing collections, along with morphological description, were found to be diploids (**Table S3**).

### 3.2 Diversity analyses of the plum species

#### 3.2.1 Chloroplast DNA markers analysis

The combination of three universal cpDNA primers resulted in distinctive haplotypes representing the studied accessions. Among the analysed material, eight accessions did not show clear PCR amplifications. All seven-primer pair–restriction enzyme combinations (K1K2-*Hinf I*, K1K2-*Taq I*, K1K2-*Alu I*, VL*-Hinf I*, VL*-Taq I*, HK-*Hinf I*, and HK*-Taq I*) amplified polymorphic patterns. After enzymatic digestion, ten polymorphic sites with 22 scorable bands were detected and ten haplotypes were identified (H1-H10) (**Table S5**). The most prevalent haplotype was H1, which was shared by 62 accessions (53%), followed by H4, which was present in 27 accessions (23.1%). H2, H6 and H7 included 15 (12.8%), two (1.7%), and six accessions (5.1%), respectively. The remaining haplotypes, H3, H5, H8, H9, and H10, were represented by only one genotype each. Haplotype H1 represented hexaploid material (*P. domestica*) with the exception of Blackthorn accession (42), of unknown ploidy level. Most of the reference accessions displayed affiliation to haplotype H1. Haplotypes H5 and H6 included solely accessions of hexaploid plum (*P. domestica*), whereas H2 and H3 were specific to the diploid cluster (*P. cerasifera*). Haplotype H4 was shared by these two *Prunus* species, with *P. cerasifera* (22 accessions) being the most abundant compared to the five *P. domestica* accessions. Haplotypes H7 to H10 were found only in *P. spinosa* accessions.

**Figure 1:**
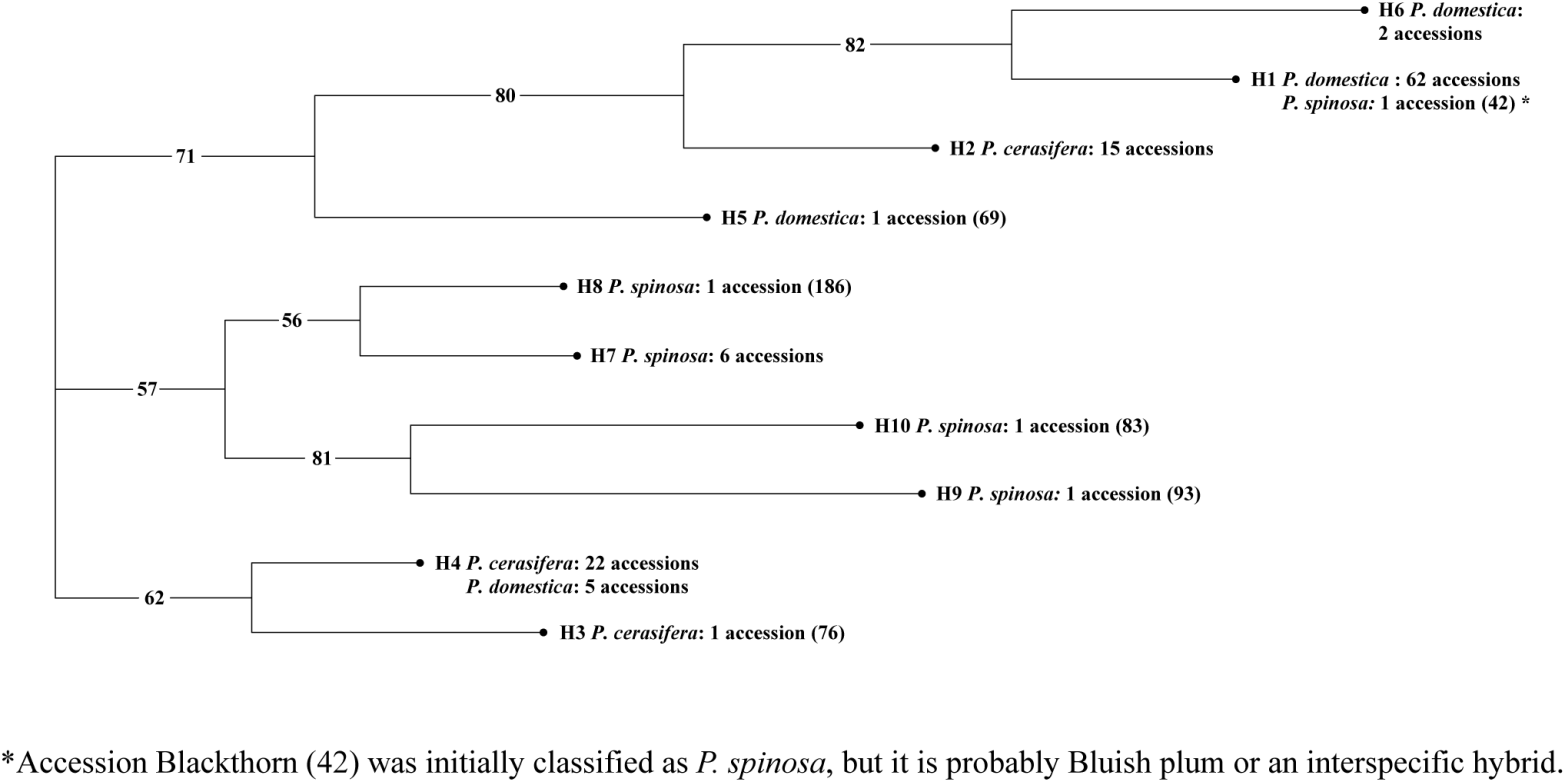
Neighbor-Joining tree based on cpDNA marker data for the three plum species (*P. cerasifera*, *P. domestica*, and *P. spinosa*). The complete list of studied accessions with the corresponding haplotypes and their codes can be found in **Table S5**.

The Neighbor-Joining tree (**Figure 1**) divided the haplotypes into three clusters supported by a high bootstrap value (>50). Cluster I was further divided into two sub-clusters, representing predominantly *P. domestica* haplotypes (H1 and H6) and haplotype H2 found only in *P. cerasifera.* Haplotype H5 consisted of only one *P. domestica* accession: ‘Pitestean’ (69), which was not positioned with the other hexaploid plums in the first sub-cluster. The cluster II grouped together all *P. spinosa* accessions, which were further subdivided into two sub-clusters, comprising haplotypes H7-H8 and H9-H10, respectively. In the cluster III, diploid plums were mainly represented (H3 and H4, in two sub-clusters). H3 was represented by only one *P. cerasifera* accession whereas sub-cluster H4 was associated with diploid plums and five hexaploid accessions: local Plum (52), ‘Empress’ (80), ‘Čačanska lepotica’ (87), ‘Quetsche du Carmel’ (169) and ‘Krikon’ (179).

#### 3.2.2 Polymorphism of SSR

Based on 11 SSR markers, the analysis revealed 116 unique genotypes. Identification of genotypes based on SSR profiles revealed the presence of three duplicate groups accounting for 6.5% (8) of the studied accessions: Plum (150) and ‘Spindel plum’ (153); common prune accessions 108 and 110; and a group of four Bluish plum accessions (112, 113, 114, and 145).

The 11 primer pairs used were polymorphic and amplified a single locus with the exception of UDP96-005, EMPAS12 and BPPCT014, which amplified two loci. The allele range for all primers extended from 131-173 (UCD-CH17) to 133-219 bp (BPPCT025), whereas the microsatellite markers (UDP96-005, EMPAS12 and BPPCT014), which amplified two loci each, had a range of alleles of 94-174 bp, 94-156 bp and 187-283 bp, respectively (**Table 2**).

**Table 2:** Distribution of SSR alleles in plum species (*P. domestica*, *P. cerasifera* and *P. spinosa*).

SSR	BPPCT014_1*	BPPCT014_2*	BPPCT025	BPPCT034	CPPCT006	CPSCT026	EMPAS06	EMPAS11
Linkage group	5	5	6	2	8	7	4	5
Total number of alleles	11	27	40	30	22	29	31	23
Number of alleles found in all three species	4	0	9	6	6	8	7	9
Range of alleles *P. domestica*	187-209	213-283	149-219	215-273	156-224	161-221	205-261	57-103
Total number of alleles in *P. domestica*	9	26	32	26	22	23	26	23
Number of alleles specific to *P. domestica*	0	16	15	2	6	7	11	5
Range of alleles *P. cerasifera*	189-199	0	133-197	217-257	172-202	171-211	179-245	57-79
Total number of alleles in *P. cerasifera*	5	0	18	11	7	17	13	9
Number of alleles specific to *P. cerasifera*	1	0	6	1	0	5	1	0
Range of alleles *P. spinosa*	187-209	211-251	143-193	211-263	174-210	171-215	197-254	57-99
Total number of alleles in *P. spinosa*	9	11	16	15	15	13	15	17
Number of alleles specific to *P. spinosa*	1	1	2	3	0	0	3	0
Number of alleles common to *P. domestica* and *P. cerasifera*	1	0	3	3	1	2	4	9
Number of alleles common to *P. domestica* and *P. spinosa*	5	10	1	5	9	3	4	17
Number of alleles common to *P. cerasifera* and *P. spinosa*	0	0	0	0	0	1	1	9
SSR	EMPAS12_1*	EMPAS12_2*	EMPAS14	UCD-CH17	UDP96-005_1*	UDP96-005_2*	TOTALS	MEAN
Linkage group	3	3	5	4	1	1	/	/
Total number of alleles	14	18	22	22	18	21	328	29.82
Number of alleles found in all three species	8	5	4	5	5	10	81	24.7
Range of alleles *P. domestica*	94-120	122-156	165-199	131-173	94-130	132-174	/	/
Total number of alleles in *P. domestica*	13	18	13	21	18	20	290	26.36
Number of alleles specific to *P. domestica*	3	5	1	4	3	4	83	28.62
Range of alleles *P. cerasifera*	94-118	122-148	171-219	135-167	96-118	132-174	/	/
Total number of alleles in *P. cerasifera*	9	10	9	12	9	13	142	12.9
Number of alleles specific to *P. cerasifera*	0	0	3	1	0	0	18	12.68
Range of alleles *P. spinosa*	94-118	122-154	167-207	139-171	100-130	140-172	/	/
Total number of alleles in *P. spinosa*	10	7	16	10	11	10	175	15.9
Number of alleles specific to *P. spinosa*	0	0	4	0	0	0	15	8.57
Number of alleles common to *P. domestica* and *P. cerasifera*	0	5	4	6	4	7	49	15.65
Number of alleles common to *P. domestica* and *P. spinosa*	1	2	6	10	6	4	83	26.77
Number of alleles common to *P. cerasifera* and *P. spinosa*	1	0	2	5	0	1	20	8.16

* Primers pairs of UDP96-005, EMPAS12 and BPPCT014 amplified two loci.

The allelic richness, generated by the 11 polymorphic primers, varied from 22 (UCD-CH17, EMPAS14, CPPCT006) to 40 (BPPCT025) alleles per locus. The total number of alleles for all accessions was 328 with an average of 29.82 alleles per locus (**Table 2**). Within identified alleles, 81 were present in the three studied species. The number and range of alleles was highest in *P. domestica*, followed by *P. spinosa* and *P. cerasifera*. The distribution of unique/specific alleles was the richest for *P. domestica* accessions (28.6%), while *P. cerasifera (*12.7%) and *P. spinosa* (8.6%) had lower values. We also calculated alleles found in only two species: *P. domestica* and *P. spinosa* shared 83 alleles; 49 alleles were found in *P. domestica* and *P. cerasifera*, while *P. cerasifera* and *P. spinosa* had only 20 alleles in common (**Table 2**).

### 3.3 Genetic structure analyses of the plum species

**Figure 2:**
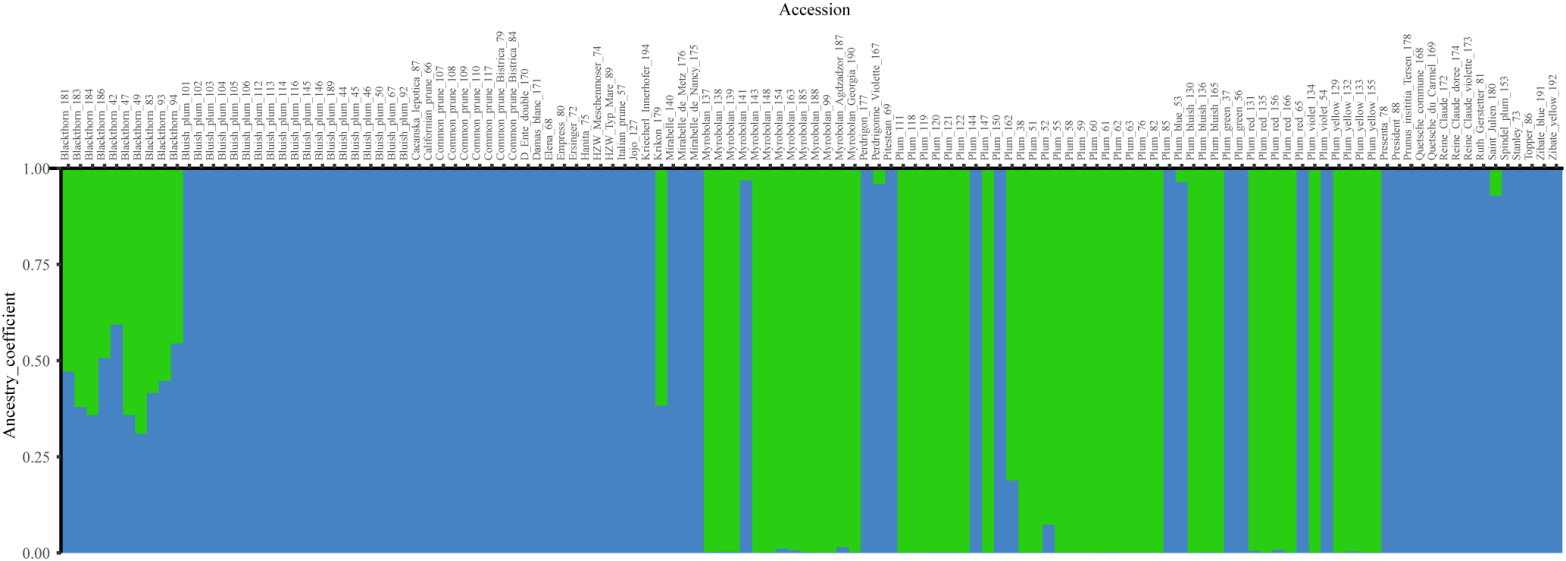
Bar plot of Bayesian analysis results (K = 2) for plum species genotypes. *P. cerasifera* accessions are shown in green (Cluster 1), and *P. domestica* accessions are arranged in blue (Cluster 2). *P. spinosa* accessions were admixed.

Bayesian analysis was used to examine the structural patterns of the three species. Regarding the most likely number of clusters (K) identified using the Evanno method, the maximum value for ΔK was two (1213.8, **Figure S4**, **Table S6**), corresponding to two ancestral populations (**Figure 2**). With a threshold of qI ≥ 0.90, 42 accessions representing diploid plums *(P. cerasifera*) were assigned to Cluster 1 (green), with the exception of the hexaploid accession Plum (52). Sixty-nine *P. domestica* accessions were assigned to Cluster 2 (blue), which were mainly identified as hexaploid, with the exception of the accessions: ‘Stanley’ (73), and Plum (144), which were found to be diploid in flow cytometry analysis. In addition, twelve genotypes showed mixed ancestry (membership values lower than 90% in both clusters). Among the admixed accessions, the highest number of accessions (10) belonged to *P. spinosa* with the remaining two genotypes representing a diploid accession, Plum (162) and one hexaploid, ‘Krikon’ (179).

**Figure 3:**
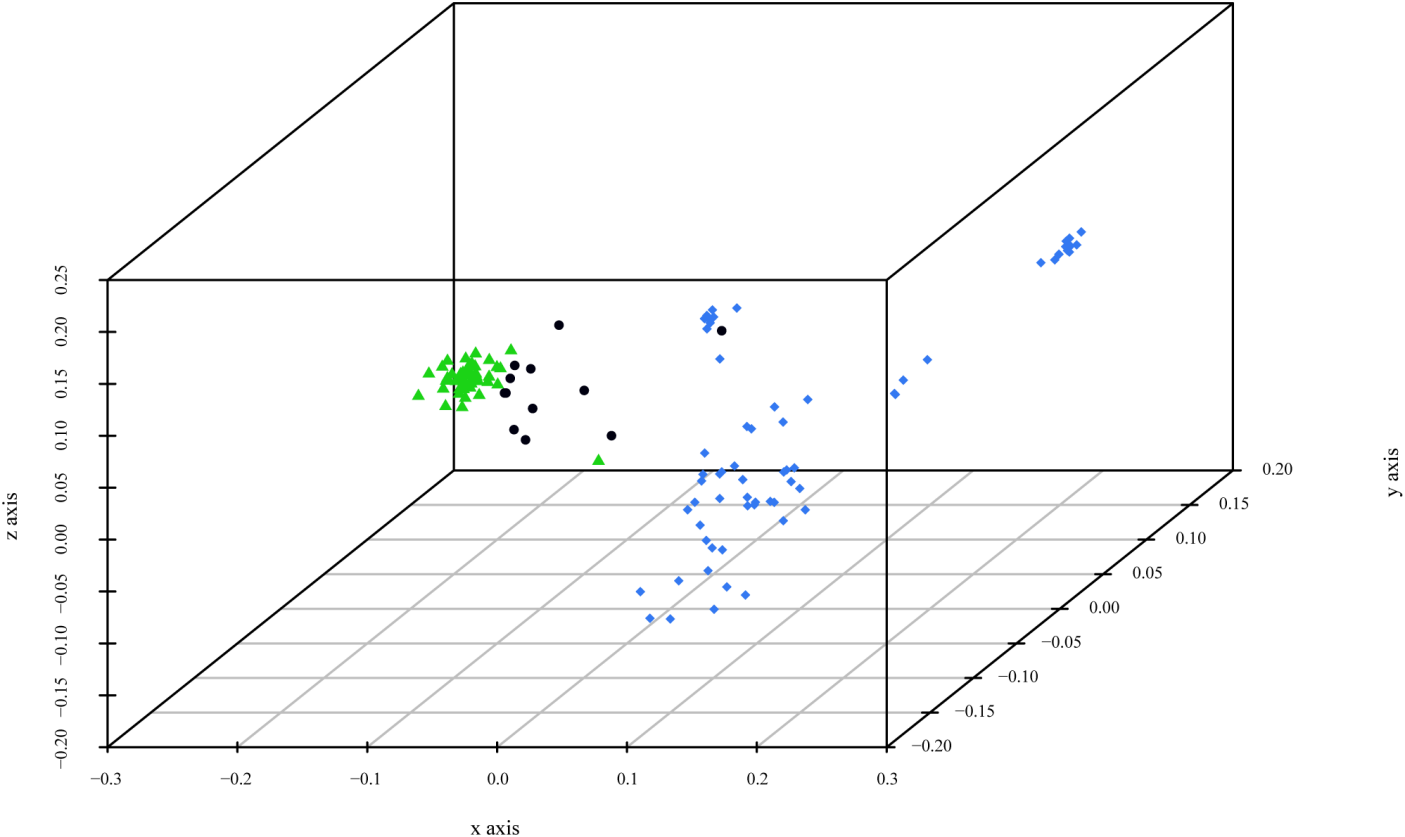
3D graph of Principal Coordinate Analysis (PCoA) based on polymorphism at 11 SSR loci of the 124 genotypes. The colors of the accessions indicate the most relevant parameter K = 2: green for *P. cerasifera* accessions, blue for mainly *P. domestica* material, and black for admixed accessions.

Before performing the Principal Coordinate Analysis, dissimilarities were estimated using two distinctive indices: Sokal and Michener index and Jaccard coefficient. Comparison of the results showed no significant differences between the two indices, and only the results of the first index are presented. PCoA analysis revealed three main clusters (**Figure 3**). Cluster 1, corresponding to *P. cerasifera*, formed a denser arrangement in space. In contrast, Cluster 2, corresponding to *P. domestica* accessions, was more dispersed. The admixed accessions (mainly *P. spinosa*) were distributed between the two clusters but closer to Cluster 1 (*P. cerasifera*).

Based on the results of various analyses (flow cytometry, cpDNA, Structure, PCoA and Neighbor-Joining), thirty-one accessions were identified, among the material collected in Slovenia, that were assumed to be hexaploid *P. domestica* but actually belonged to the diploid species *P. cerasifera*.

### 3.4 Genetic structure analyses and relationships of *P. domestica*

One of the main objectives of this study was to assess the genetic structure of *P. domestica* collected in Slovenia.

**Figure 4:**
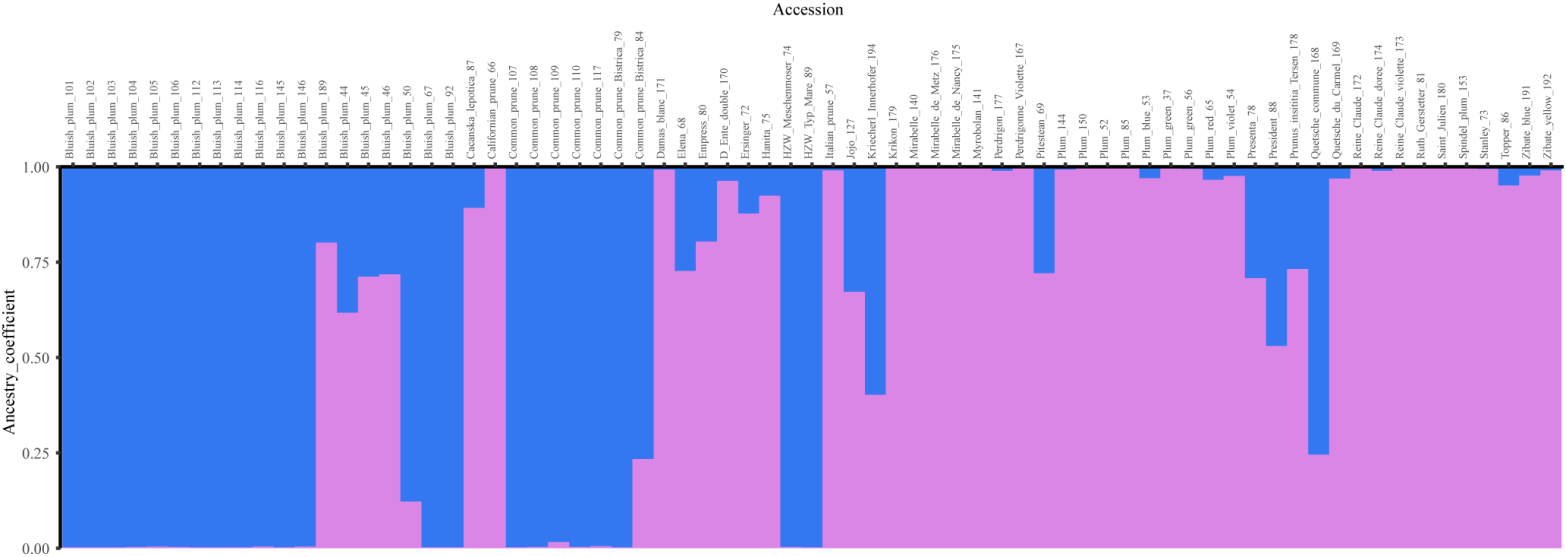
Bar plot of Bayesian analysis results (K = 2) for *P. domestica* accessions. Twenty-two accessions representing mainly local traditional material collected in Slovenia, were assigned to the Cluster 1 (blue). Cluster 2 (violet) consisted of 32 accessions of mixed origin (*in situ* and *ex situ*) representing different pomological groups. Seventeen accessions were classified admixed.

When investigating patterns of structure among *P. domestica* accessions with Bayesian analysis, two ancestral populations were identified using the ΔK method (**Figure S5**, **Table S7**). The threshold value of qI ≥ 0.90 assigned twenty-two accessions to the Cluster 1 (blue). They represented mainly the local traditional *P. domestica* accessions of Bluish plum and common prunes collected in Slovenia, and additionally two traditional German prunes: ‘HZW Meschenmoser’ (74) and ‘HZW typ Mare’ (89) (**Figure 4**). Cluster 2 (violet) consisted of 32 accessions of mixed origin, including: five accessions collected *in situ* in Slovenia, Austria and Croatia; four improved cultivars [‘Stanley’ (73), ‘Hanita’ (75), ‘Ruth Gerstetter’ (81) and ‘Topper’ (86)]; eleven accessions from the SPGB collection, the Botanical Garden of the University of Maribor, the Botanical Garden of the Karl-Franzens University of Graz, and twelve accessions from the PFNC collection. The latter are reference accessions representing different pomological groups (such as mirabelles and greengages), some French landraces [such as ‘Quetsche du Carmel’ (169), ‘D’Ente double’ (170), ‘Damas blanc’ (171), ‘Perdrigon’ (177), ‘St. Julien’ (180)], and genotypes such as damson ‘Krikon’ (179). Seventeen accessions with estimated membership coefficients below 0.9 were considered admixed, including five accessions from Bluish plum group (44, 45, 46, 50 and 189). The average distance (expected heterozygosity) between individuals was higher in Cluster 2 (0.8627) than in Cluster 1 (0.6617).

Examining the structure of the plum collection for K ranging between 3 and 6 (**Figure S6A–D**) allowed us to detect interesting dynamics among the different plum groups. At K=3 (**Figure S6A**), Cluster 1 split into two sub-clusters, Bluish plum group (olive green) and the common prunes (blue), while the original Cluster 2 (violet) and admixed accessions maintained the same profile or remained unchanged, except for the accession ‘Kriecherl Innerhofer’ (194), which shared a large part of its genome with the Bluish plum group. When K=4 (**Figure S6B**) was considered, 14 accessions (red) separated from Cluster 2 (K=2). They corresponded to known pomological groups such as mirabelle plums (140, 175, 176) and greengage accessions (172, 173, 174), as well as ‘Damas blanc’ (171), ‘St. Julien’ (180) and ‘Italian prune’ (57). At K=5 (**Figure S6C**), Bluish plum accessions 44, 45, 46 (before admixed) formed a new cluster (yellow) that also included accessions Plum (150) and ‘Spindel plum’ (153). At K=6 (**Figure S6D**), Plum (150) and ‘Spindel plum’ (153) were associated with five other accessions: ‘Plum red’ (65), ‘Stanley’ (73), ‘Čačanska lepotica’ (87), ‘President’ (88) and ‘Jojo’ (127), forming an additional ancestral population (cyan).

**Figure 5:**
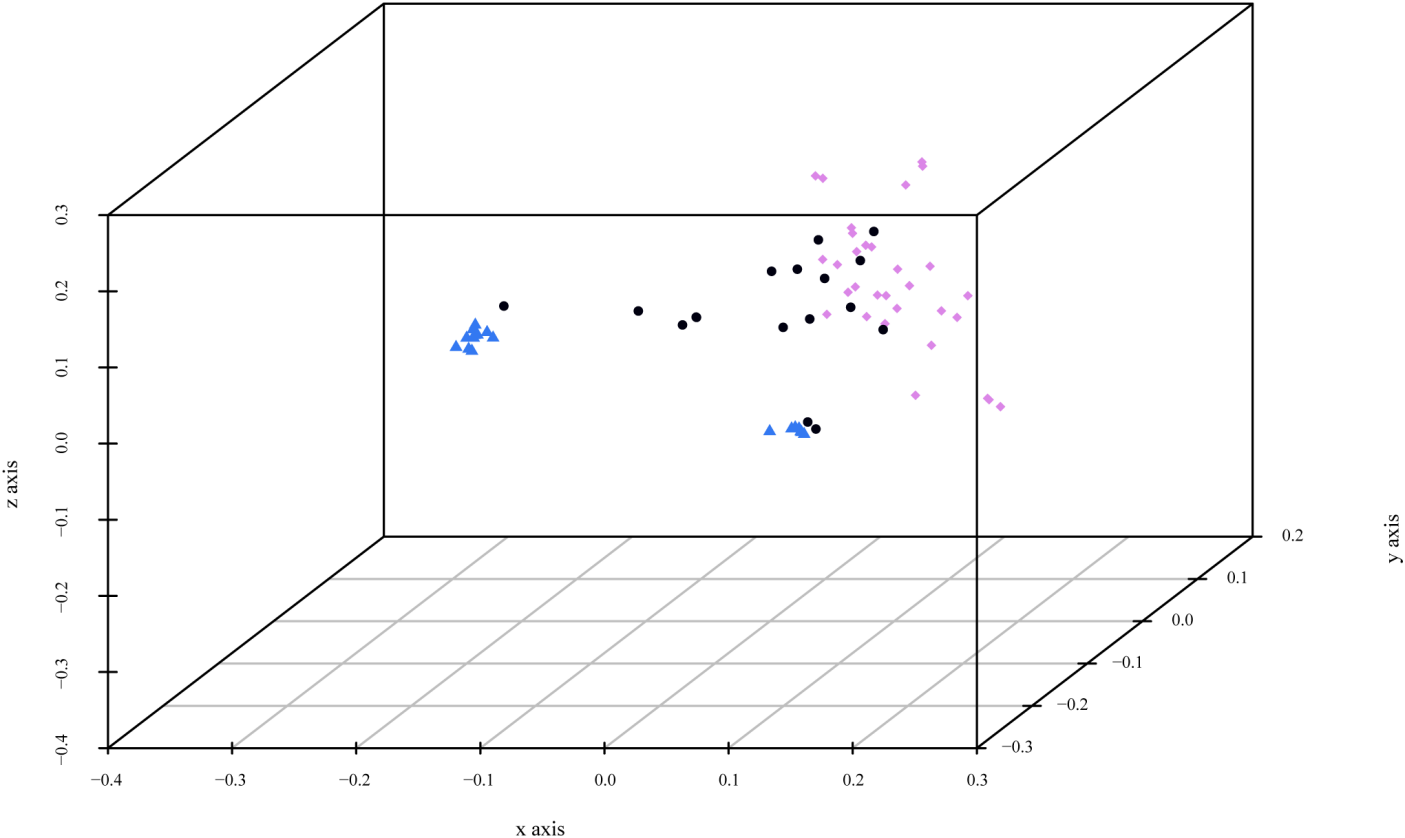
3D graph of Principal Coordinate Analysis (PCoA) based on polymorphism at 11 SSR loci of the 71 P*. domestica* genotypes. The colors of the accessions indicated the clusters obtained by Bayesian analysis for K=2: Bluish plum accessions and common prunes collected in Slovenia (blue), plums genetically very diverse (violet). Accessions located between the two clusters (in black) correspond to the admixed accessions.

PCoA confirmed the results obtained with Structure for K=2 (**Figure 5**). Cluster 1 was represented by Bluish plum accessions and common prunes collected in Slovenia. Cluster 2 consisted of 32 genetically very diverse materials: some plum accessions collected *in situ*, international improved cultivars, and reference accessions (described in detail above). It was more dispersed and divided into several sub-clusters, demonstrating a higher genetic diversity. The accessions located between the two clusters corresponded to the admixed accessions identified by the Bayesian analysis.

**Figure 6:**
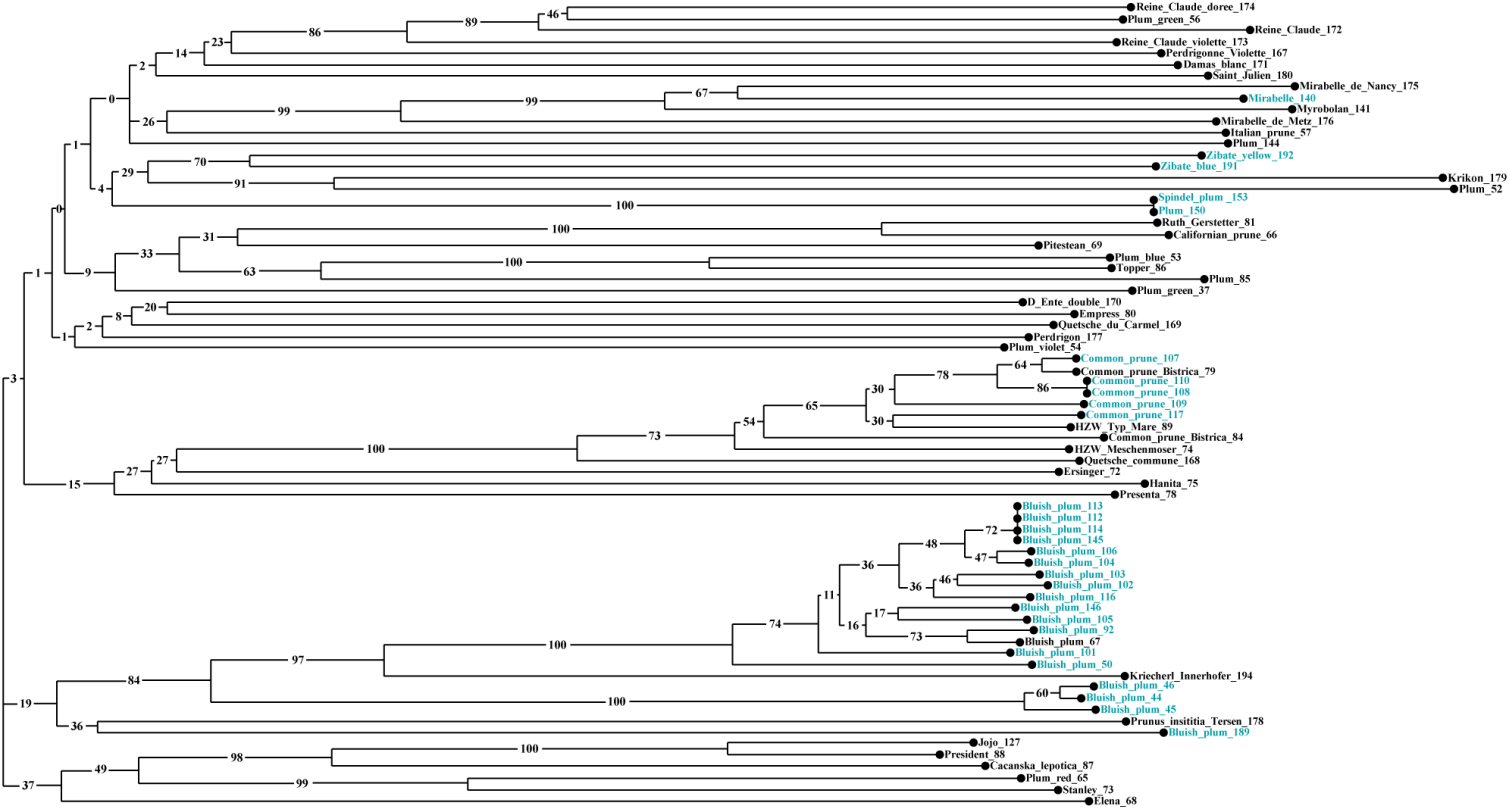
Neighbor-Joining tree based on the dissimilarity matrix calculated from the data set of 11 SSRs for 71 P*. domestica* accessions using the Sokal and Michener index. The colors of the accessions reflect the type of studied material: *in situ* material (blue) and *ex situ* material (black).

Neighbor-Joining analysis allowed us to evaluate the genetic relationships between 71 P*. domestica* accessions (**Figure 6**, **Figure S7**). Three clusters were observed in the dendrogram. Cluster I was the greatest (44 accessions), quite complex and divided into two sub-clusters. The first sub-cluster included plum accessions that were very different morphologically. This sub-cluster could be related to Cluster 2 identified by Bayesian analysis. First, all French greengages were established: ‘Reine Claude’ (172), ‘Reine Claude Violette’ (173), and ‘Reine Claude Dorée’ (174), together with an improved cultivar, ‘Plum green’ (56), from Slovenia. The French landraces ‘Perdrigonne Violette’ (167), ‘Damas Blanc’ (171) and ‘Saint Julien’ (180) were associated to this group, but with low bootstrap values. In the second group, two Slovenian accessions, Mirabelle (140) and supposed Myrobolan (141), clustered very strongly with French mirabelle reference cultivars, e.g., ‘Mirabelle de Nancy’ (175) and ‘Mirabelle de Metz’ (176). Two accessions were placed near the mirabelle group, Plum (144) and an ‘Italian Prune’ (57). Below, two pairings stood out: ‘Zibate blue’ (191) and ‘Zibate yellow’ (192), native small-fruited subsp. *insititia* genotypes from Austria; ‘Krikon’ (179) clustered together with a local accession Plum (52). At the end of the first part of this sub-cluster, a pair of duplicate genotypes, Plum (150) with ‘Spindel plum’ (153), was found. The second half of the first sub-cluster consisted mainly of well-known improved cultivars: ‘Ruth Gerstetter’ (81), which appeared to be genetically close to the landrace ‘Californian Prune’ (66), ‘Pitestean’ (69), and ‘Topper’ (86). Associated with the latter were an improved variety ‘Plum Blue’ (53) maintained in the SPGB and a very old variety from Croatia, Plum (85), which is a prune type. The Slovenian small fruited genotype ‘Plum Green’ (37) was connected to this sub-cluster, although with a low bootstrap value. The second sub-cluster of Cluster I consisted of common prunes, collected *in situ* in Slovenia, well-known cultivars such as ‘Bistrica’ (79, 84) or ‘Quetsche commune’ (168), or derived from clonal selection [‘HZW Meschenmoser’ (74) and ‘HZW Typ Mare’ (89)] or from crosses involving common prunes [‘Ersinger’ (72), ‘Hanita’ (75) and ‘Presenta’ (78)].

The Cluster II included all accessions of the Bluish plum group, separated into two groups according to the results of Bayesian analysis. One corresponded to Structure Cluster 1 and the other combined the accessions previously identified as admixed in the genetic structure analysis. The first group included material from different locations in the Styria region, associated to them, but clearly separated, was accession ‘Kriecherl Innerhofer’ (194) from Austria. The second group, much smaller, consisted of three accessions collected in the Prekmurje region. Related to both groups were found a *Prunus insititia* accession ‘Tersen’ (178), originating from Sweden and maintained in the PFNC collection, and the last Bluish plum (189), collected in eastern Slovenia. Cluster III contained several very well-known modern cultivars such as ‘Elena’ (68), ‘Čačanska lepotica’ (87), ‘President’ (88), ‘Jojo’ (127) and ‘Stanley’ (73).

## 4 Discussion

### 4.1 Relationship among *P. spinosa*, *P. cerasifera* and *P. domestica* species

In this study we tried to collect mainly *P. domestica* accessions/germplasm, but we discovered that approximately a third of the collected material actually belonged to *P. cerasifera*. This surprising result might be related to the fact that the Slovenian term ‘ringlo’, traditionally used for greengages, can also refer to any plum that is round, which is a typical characteristic of *P. cerasifera* fruits. When we carried out the initial prospection, we did not aim at species identification, but at categorizing accessions based on fruit description or other interesting agronomic traits.

Assessment of genetic diversity using PCR-RFLP analyses showed that the total number of haplotypes (10) was lower compared to similar studies: *P. spinosa* (4), *P. domestica* (3), *P. cerasifera* (2), and, as mentioned above, one haplotype was shared by the latter two species. Horvath et al. (2011) discovered the highest total number of haplotypes (32): *P. cerasifera* (15), *P. spinosa* (12) and *P. domestica* (5). On the other hand, Urrestarazu et al. (2018) reported the highest number of *P. domestica* haplotypes (8), which is consistent with the fact that they studied the highest number of *P. domestica* accessions (166). The most predominant haplotype in our study contained mainly *P. domestica* accessions. Urrestarazu et al. (2018) also reported the presence of a predominant haplotype, whereas two major haplotypes were identified in the case of Horvath et al. (2011). Both authors argue that a reduction in cpDNA haplotypes of *P. domestica* could be related to historical events (geological events, climatic oscillations, and independent dispersal of the species across continents), human interference and a narrow genetic base of material when plum was introduced into Europe. However, the relatively small number of accessions in our study could also explain this observation. Concerning *P. spinosa*, Horvath et al. (2011) observed more complex clustering because although most haplotypes of *P. spinosa* clustered together (8), four were linked to haplotypes of *P. domestica* and *P. cerasifera*. Because several identical accessions were examined in both studies (21) and the same primer pair–restriction enzyme combinations were used for cpDNA marker analysis, this discrepancy is rather unexpected. For 17 accessions, we confirmed the same trend for the corresponding haplotypes in both studies. However, three *P. spinosa* (181, 183, 184) accessions did not form individual haplotypes as was the case in Horvath et al. (2011). We suspect that this discrepancy is due to the difference in resolution of the amplified products.

When studying SSR markers polymorphism, we obtained an average value of 29.82 alleles per locus, similar to the value of 29.25 alleles for eight SSR loci reported by Gaši et al. (2020). In contrast, Horvath et al. (2011) found an average of 41 alleles for five loci, while Urrestarazu et al. (2018) determined an average of 23.36 alleles after analysing 11 SSRs. Slightly lower values of 22.7 and 18.7 alleles for nine SSR loci were determined by Sehic et al. (2015) and Kazija et al. (2014), respectively. These studies differ in the evaluated material. While Sehic et al. (2015), Urrestarazu et al. (2018), and Gaši et al. (2020) studied exclusively *P. domestica* accessions, Horvath et al. (2011) and our work included accessions of *P. domestica*, *P. cerasifera* and *P. spinosa* species. Our results showed lower diversity than those of Horvath et al. (2011), which could be due to the fact that our study focused more on local landraces such as the Bluish plum group and common prunes from Slovenia, while Horvath et al. (2011) included material from different countries across Europe, especially for the *P. spinosa* and *P. cerasifera* species.

When comparing all three species, the number, range of alleles, and distribution of unique/specific alleles were highest for *P. domestica*, which is not surprising given its polyploid nature as well as the largest number of accessions studied. The number of *P. spinosa* accessions for this study was the lowest of all three species, nevertheless the number and range of alleles were higher compared to *P. cerasifera.* This demonstrates the originality of the collected *P. spinosa* material.

In addition, cpDNA analysis complemented with SSR markers allowed us to reveal some examples of complex relationships between the three species. Firstly, the supposedly *P. spinosa* accession Blackthorn (42), shared haplotype H1 with *P. domestica* material. Furthermore, the results of a Neighbor-Joining analysis for the three species (data not shown) indicated that Blackthorn (42) clustered together with a group of Bluish plum accessions (44, 45, and 46). However, Bayesian analysis classified this accession as admixed, together with other *P. spinosa* accessions. Hence, genetic relationship analyses suggest that Blackthorn (42) accession could be either a Bluish plum or an interspecific hybrid. Secondly, ‘Krikon’ (179) is another accession that exhibited a putative hybrid origin. When all species were examined together (data not shown), ‘Krikon’ clustered with the *P. cerasifera* and *P. spinosa* material. Interestingly, Zhebentyayeva et al. (2019) found that the same ‘Krikon’ accession from the PFNC collection clustered close to a *P. cerasifera* accession from the UC Davis collection and a *P. spinosa* accession from Sweden.

### 4.2 Involvement of *P. cerasifera* and *P. spinosa* in *P. domestica* origin

The combined results presented in this study allowed us to investigate the potential involvement of *P. cerasifera* and *P. spinosa* species in the origin of *P. domestica.* Different analyses provided somehow contradictory information, which is not surprising given the unresolved question of the genetic origin of *P. domestica*. Assessment of genetic diversity using chloroplast DNA markers showed that *P. spinosa* clearly clustered separately, whereas *P. domestica* and *P. cerasifera* clustered together. The haplotypes of *P. domestica* and *P. cerasifera* appeared to be genetically closer compared to the haplotypes of *P. spinosa*, suggesting that *P. cerasifera* may have contributed to the maternal chloroplast DNA of *P. domestica*. This was also discussed by Bortiri et al. (2009), Reales et al. (2009), and Horvath et al. (2011). On the contrary, SSR markers revealed a possible involvement of *P. spinosa* in the genome of *P. domestica*, sharing almost twice as many alleles as *P. domestica* with *P. cerasifera*, which is consistent with results of Horvath et al. (2011). This could support a hybrid origin of the European plum. Moreover, the genetic structure analyses revealed *P. domestica* and *P. cerasifera* as independent groups, while the accessions of *P. spinosa* showed admixed ancestry shared with both species.

### 4.3 Genetic structure and relationships between *P. domestica* accessions

In this study, different approaches allowed us to establish genetic relationships among the local plum material collected in Slovenia and the traditional plum groups, such as common prunes, greengages and mirabelles.

Duplicates, represented 11.3% of the studied *P. domestica* accessions. Kazija et al. (2014) found a similar proportion of duplicates (11%), when they examined the plum germplasm in Croatia. The percentage of identified duplicates was slightly higher in Urrestarazu et al. (2018) at 18%, while Sehic et al. (2015) detected 30% duplicates.

The STRUCTURE results were confirmed by the representation of PCoA analysis based on a genetic distance matrix. The studied *P. domestica* accessions formed two distinct ancestral populations, which was also reported for plum by Gaši et al. (2020), as well as for other germplasm collections of important fruit tree species such as apple (Urrestarazu et al., 2012), cherry (Campoy et al., 2016), walnut (Bernard et al., 2018) and apricot (Bourguiba et al., 2020). The average distance between individuals (expected heterozygosity) was higher in Cluster 2, which could be explained by the diverse origin of the accessions belonging to this cluster (landraces from the different pomological groups, accessions from botanical gardens, and international modern cultivars) and by their higher number. On the contrary, Cluster 1 consisted mainly of two plum groups (Bluish plum group and common prunes) collected in Slovenia.

The landraces belonging to the Bluish plum group are historically and genetically important plums that are slowly disappearing from the Slovenian environment. Since there is very scarce information about this plum group (except in oral tradition), we have tried to find reference material, corresponding to the morphological description of this varietal group in order to link them with some known genetic material. The French landrace ‘Perdrigon’ is a small, round-fruited, bluish-purple plum that was cultivated in Slovenia in the 19^th^ century (Zalokar, 1854). However, two accessions of this landrace group, ‘Perdrigonne Violette’ (167) and ‘Perdrigon’ (177), as well as the accession ‘Saint Julien’ (180), which are morphologically similar to the description of Bluish plum landraces, were not genetically close to the Bluish plum group. The same is true for ‘Krikon’ (179), which is probably of Swedish origin and represents a group of sour-tasting feral plums with small, round, and bluish (bluish-red) fruits (Sehic et al., 2015). On the other hand, *Prunus insititia* ‘Tersen’ (178), which also originates from Sweden, clustered with several Bluish plum accessions. The fruits of ‘Tersen’ are small, blue-black in colour, and traditionally used for jams. Finally, ‘Kriecherl Innerhofer’ (194) is a clonal selection within subsp. *insititia*, a small, round, bluish-purple coloured landrace from Styria region in Austria. It is traditionally used for the production of plum brandy. Because its geographic origin, morphological appearance, and use overlap with our Bluish plum landraces, we suspected that they might be genetically close, which was confirmed by our analyses. The SSR analyses divided the Bluish plum accessions into two distinct populations, which were established in agreement with the region of origin. Similar to the first population, the Bluish plum accession (189) was also collected in the same region (Styria), although approximately 75 km of air distance apart and 100 m difference in elevation. It was in the same cluster with other Bluish plum populations, but separated from them and connected with *Prunus insititia* ‘Tersen’ (178). Since it seems to be a rather original material, it should be further investigated, by studying a larger sample at the same location. While Mišić (1979) described Bluish plum to be one of the widespread landraces in Slovenia, this did not correspond to our experience while collecting material. Bluish plum has low to medium susceptibility to some important plum diseases like *Plum pox virus*, *Monilia laxa* and *Monilia fructigena*. Perhaps this is the reason why they are slowly disappearing from the natural environment. Nevertheless, it might be interesting to test genetically well-identified Bluish plum accessions for tolerance to these diseases. There is also a possibility that Slovenian fruit producers and local farmers have tried to introduce more modern cultivars in recent years and somehow ‘abandoned’ the traditional material.

Another important locally collected group of plums are the common prunes, which clearly formed a unique group and seemed to include similar, but not identical accessions. When structure was examined (K=3), common prunes originating from different parts of Europe, such as Slovenia, France or Germany, formed a separate cluster, which was also confirmed by the Neighbor-Joining analysis. This result corroborates the fact that common prunes are widespread in Europe, with a large number of types that are distributed under different names such as ‘Hauszwetsche’ (Germany), ‘Quetsche Commune’ (France), ‘Casalinga’ (Italy), ‘Besztercei’ (Hungary) and ‘Vinete romanesti’ (Romania) (Mišić, 1979). Linked by parentage to the common prune cluster were early selections or modern cultivars: ‘Ersinger’ (72), ‘Hanita’ (75) (‘President’ × ‘Auerbacher’) and ‘Presenta’ (78) (‘President’ × ‘Ortenauer’). The results on the cultivars ‘Hanita’ and ‘Presenta’ could appear somewhat controversial since, according to the NJ analysis, they are closer to the common prunes, than to the female parent, ‘President’. The male parents, respectively ‘Auerbacher’ and ‘Ortenauer’, are random common prune seedlings (Hartmann and Eckhart, 2008). However, the Bayesian analysis showed the connection to the cultivar ‘President’, which they have as common genitor. Some of the well-known common prunes such as ‘Italian prune’ (57), ‘Californian prune’ (66), or ‘Quetsche du Carmel’ (169) did not group in any of the above clusters. For the latter, cpDNA markers analysis revealed a different haplotype (H4) than in most of *P. domestica* accessions (H1), which was also pointed out by Horvath et al. (2011). The accessions ‘Italian’ and ‘Californian’ prune in this study were named by the grower and therefore may not be true representatives of this plum group.

Genetic analyses highlighted the specific position of greengages and mirabelles compared to the other plums. Structure analysis considering K=4, showed that these two pomological groups share a common ancestry. Horvath et al. (2011) revealed the admixed origin of the material of both pomological groups, which was not the case in our study. Regarding specifically the greengage group, several authors noted that this pomological group is well differentiated (Horvath et al., 2011; Urrestarazu et al., 2018; Zhebentyayeva et al., 2019), with the exception of the study by Gaši et al. (2020). In addition, Zhebentyayeva et al. (2019) found that greengages together with ‘Prune d’Agen’ (common prune) clearly stood out from the rest of the studied *P. domestica* germplasm, as shown by the highest fixation indexes (F_ST_) reported for these two groups. The Neighbor-Joining analysis divided the greengage and mirabelle accessions into two distinct groups associated with Cluster I. In the first group, ‘Plum green’ (56) from Slovenia was strongly linked to all French greengages. Although it was registered under a different name, genetic analyses and morphological characteristics confirmed that this plum belonged to the greengages. The second group of Cluster I was formed by two mirabelles from Slovenia: Mirabelle (140) and Myrobolan (141). The latter was initially thought to be diploid *P. cerasifera*, hence the name, but flow cytometry combined with the other genetic analyses confirmed that it actually belonged to the hexaploid mirabelle group. These two accessions showed strong affinity to French mirabelles, confirming historical reports (Zalokar, 1854). Kazija et al. (2014) and Zhebentyayeva et al. (2019) identified mirabelle accessions as a unique plum group, however results of our study are consistent with those of Horvath et al. (2011) and Sehic et al. (2015), where mirabelle plums were never identified as a distinct genetic group.

The Neighbor-Joining analysis clustered some of the modern cultivars according to pedigree relationships. For example, the Cluster III consisted of well-known cultivars such as ‘Elena’ (68), ‘Čačanska lepotica’ (87), ‘Jojo’ (127), located close to their genitor ‘Stanley’ (73). Since the latter is a well-known international cultivar belonging to *P. domestica*, it was surprising to learn that the flow cytometry results counted this accession among diploids. This result could be due to an error in the collection of material, although the results of the genetic analyses showed a well-established position with the progeny cultivars. Hence, we suspects a measurement error in the flow cytometry studies. According to some earlier/older references (Mišić, 1979), ‘Čačanska lepotica’ derived from the cross ‘Wangenheims Frühzwetsche’ × ‘Požegača’ (syn. ‘Bistrica’, represented in our work by accessions 79 and 84); however, more recent studies have shown that the supposed father is cultivar ‘Stanley’ (Heinkel et al., 2000; Decroocq et al., 2004), as shown as well in our study. Nevertheless, there are still discrepancies regarding the maternal component. While the study by Heinkel et al. (2000) supported ‘Wangenheims Frühzwetsche’ as the mother plant, Decroocq et al. (2004) stated that the supposed female genitor could be ‘Ruth Gerstetter’. In our study, ‘Čačanska lepotica’ did not cluster closely neither to the common prune (‘Bistrica’) nor to ‘Ruth Gerstetter’. Another interesting modern cultivar was ‘Pitestean’ (69) (‘Tuleu Timpuriu’ × ‘Early Rivers’), which represented a single cpDNA haplotype (H7). ‘Tuleu Timpuriu’, of Romanian origin, is a progeny of ‘Tuleu gras’, an autochthonous variety of *P. domestica* from the Subc arpathian area (Mihai et al., 2012). This result agrees with the study of Urrestarazu et al. (2018), in which ‘Tuleu gras’ was identified as a unique haplotype.

## 5 Conclusions

The combined results provide insight into the relationship between the three plum species, *P. spinosa, P. cerasifera and P. domestica*. Our data disregard the possibility that *P. domestica* was entirely derived either from *P. cerasifera* or from *P. spinosa*. We were able to confirm that *P. cerasifera* may have contributed to the maternal lineage of *P. domestica*, and we suggest the possible involvement of *P. spinosa* in the *P. domestica* genome. Regarding the origin of *P. domestica* we managed to draw some parallels with other studies, however, our data were not conclusive enough to definitely resolve between different hypotheses. To address this question, further research should include genomics strategies including sequencing of cpDNA on a larger sampling of the three species covering their probable centres of origin and domestication. In addition, investigation of the genetic diversity using S-locus genotyping (Marchese et al., 2010; Halász et al., 2017) could provide new information on the origin, geographical distribution, and occurrence of possible hybridization events between plum species.

Our study revealed very low redundancy that occurred only in *P. domestica* accessions collected *in situ*, demonstrating the authenticity of the collected material. It was surprising to discover that approximately a third of the material initially collected as *P. domestica* actually belonged to *P. cerasifera*. This shows the genetic complexity of plum species and highlights the importance of combining different tools and marker systems to evaluate relationships between the studied species.

Even though prospection of Slovenian plum germplasm was preliminary, we found valuable material collected from a relatively small area. For example, Bluish plum accessions forming two distinct populations were collected within a radius of about 100 km. It would be interesting to cover a wider area, and try to collect different genotypes and study in depth a larger variability of this local population. Another traditionally important group that could be included in a more comprehensive study is the common prunes. Moreover, this type of research could be a next step for a comprehensive evaluation of plant genetic resources in the Balkan Peninsula, which harbors potentially valuable and unique plum genotypes.

Finally, the obtained molecular data and the characterization of the collected material will be crucial in order to understand the diversity of Slovenian germplasm, to improve the conservation process, to recover local genotypes and to enrich the current SPGB collection. Furthermore, a complete agro-morphological study would be essential to evaluate prospective material in an attempt to integrate valuable genotypes into breeding programs. In addition, a thorough evaluation of commercial value for use in plum production would also be required.

## Data availability statement

The original contributions presented in the study are included in the article/**Supplementary Material**. Further inquiries can be directed to the corresponding authors.

## Author contributions

AI, MŠ, AŠ and TT designed the initial research, TB and JQ-G suggested complementary research and methods in data analysis. AI, MŠ, AŠ and TT conducted the field work and sampling. TT carried out SSR molecular analyses. TT and TB evaluated the SSR analyses results. TT analyzed the data. TB and JQ-G contributed to the data analysis. TT, TB and JQ-G, wrote the manuscript, AI, MŠ, AŠ edited and reviewed the original manuscript. All authors contributed to the article and approved the submitted version.
