# Supplementary material for: Genetic diversity and structure of Slovenian native germplasm of plum species (P. domestica L., P. cerasifera Ehrh. and P. spinosa L.)
Source: Front Plant Sci. 2023 Mar 21;14:1150459. doi: 10.3389/fpls.2023.1150459 (PMC10070851; doi:10.3389/fpls.2023.1150459)
Supplement: Supplementary file 1 [file DataSheet_1.pdf]

(A)

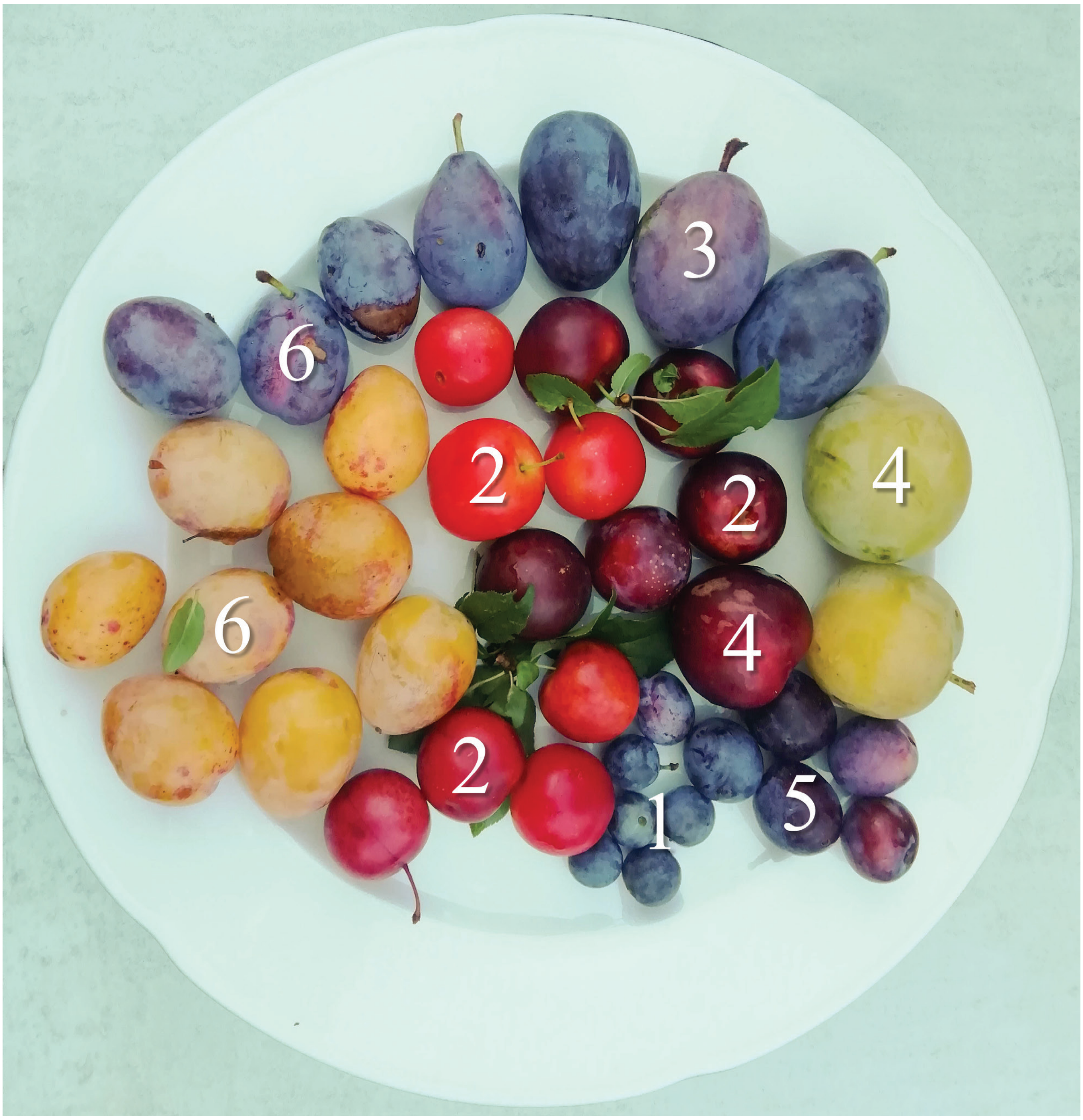

continued

(B)

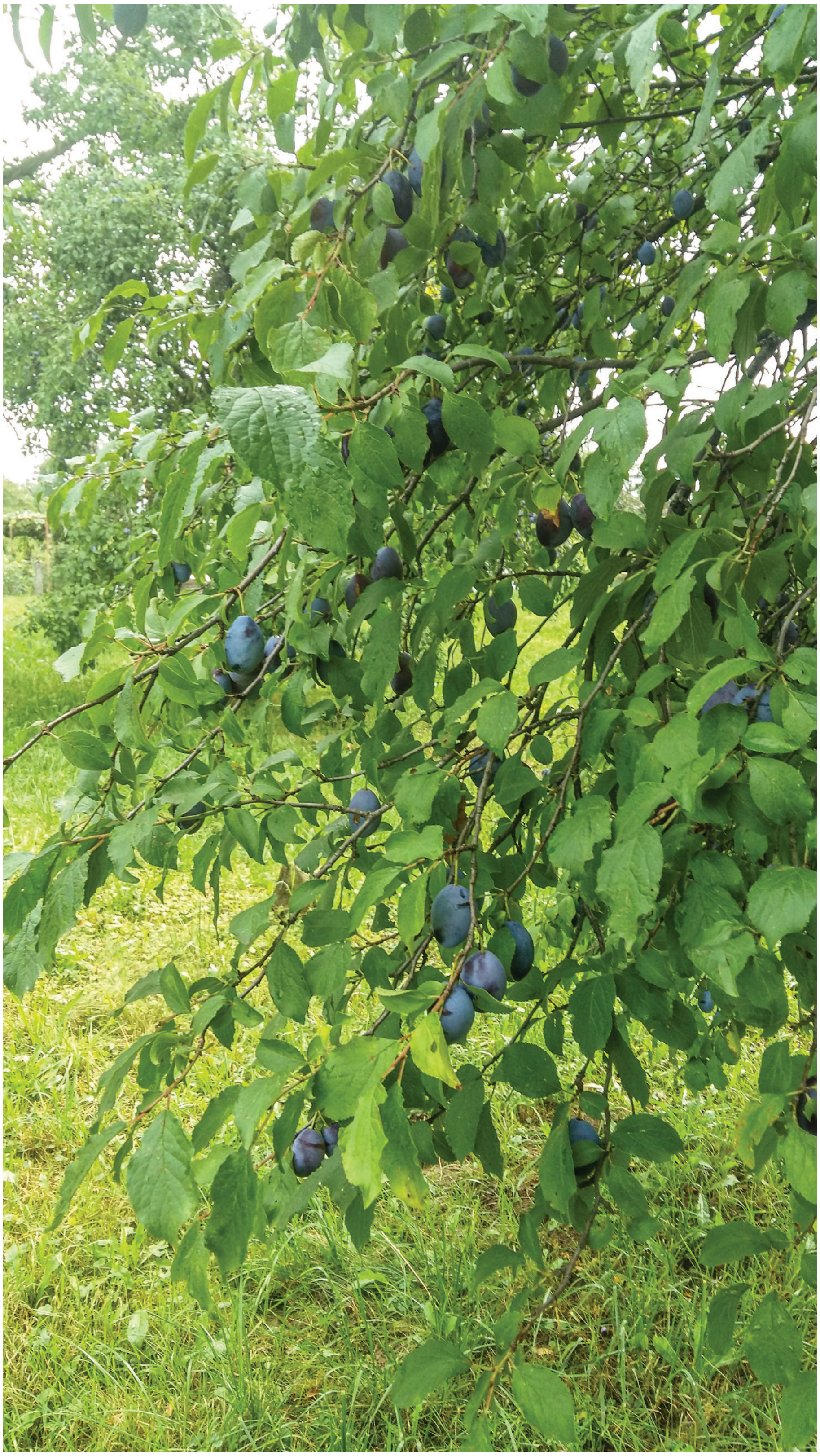

continued

(C)

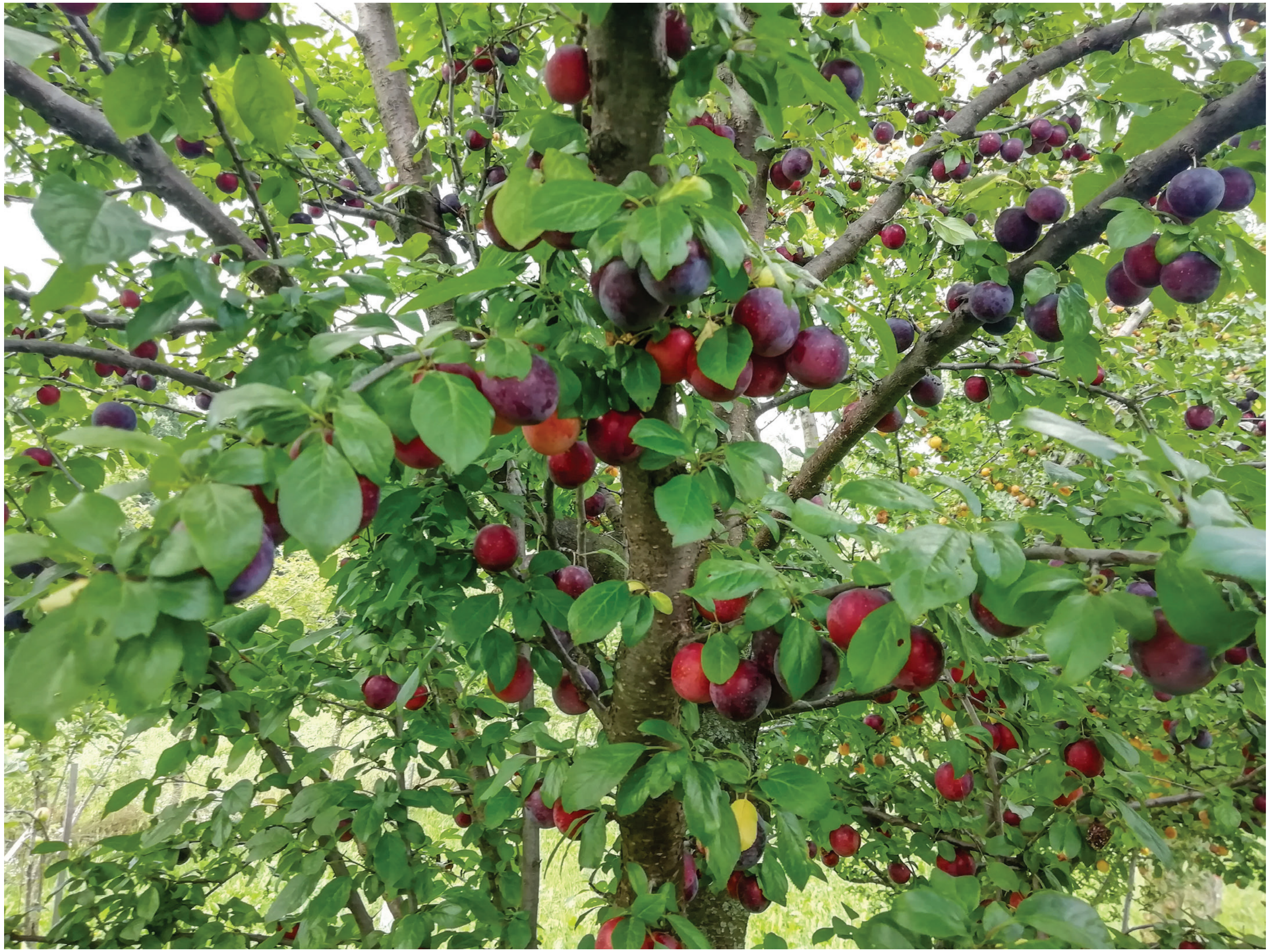

continued

(D)

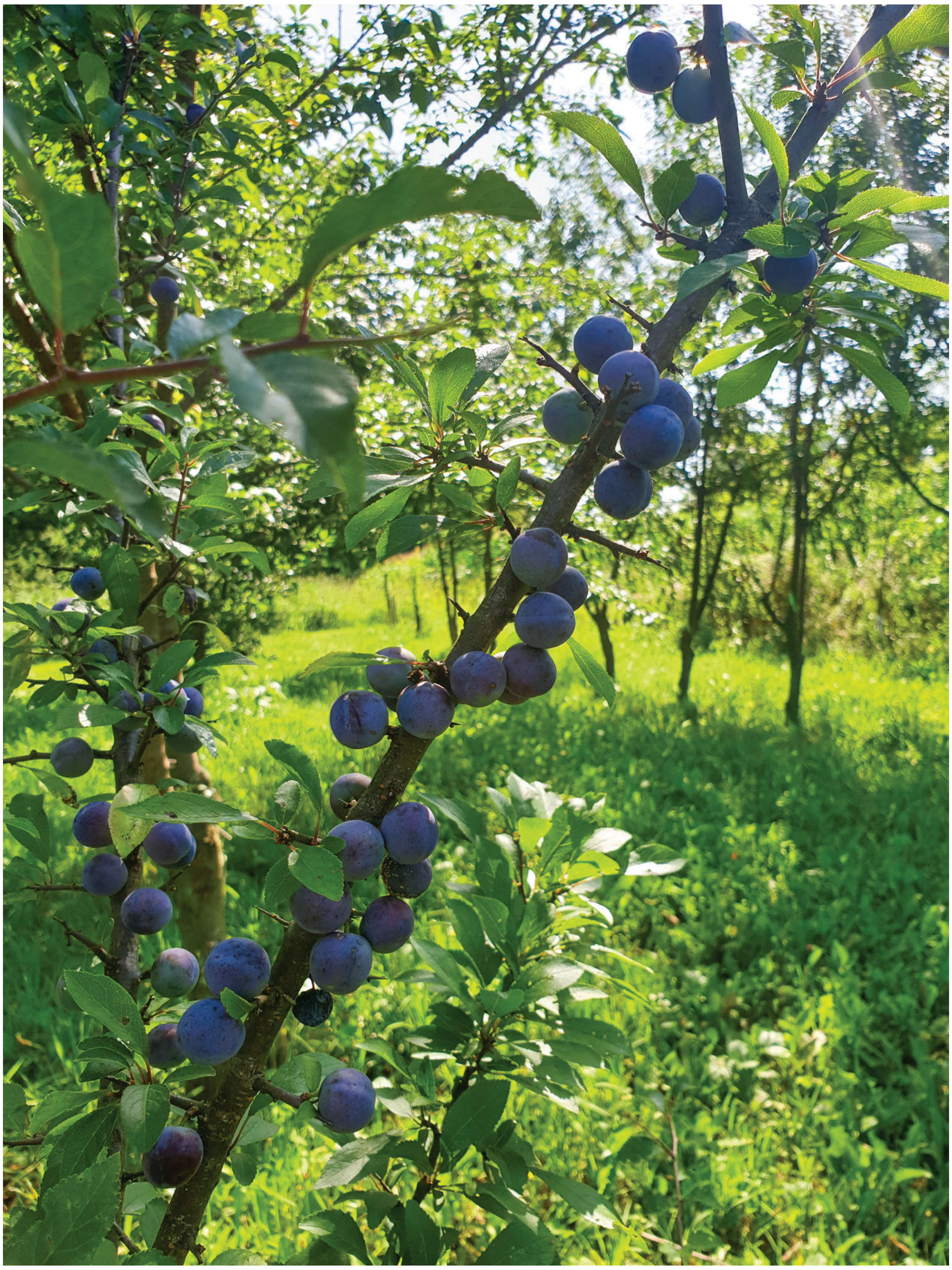

Figure S1:
A: Plum fruits of *P. spinosa*, *P. cerasifera* and of several cultivars of *P. domestica*, the picture highlights the wide variability in fruit color, size and shape (*P. spinosa:*1, *P. cerasifera:* 2, *P. domestica* : 3 modern cultivars, 4 greengages, 5 traditional Bluish plum, 6 traditional common prunes);
B: *P. domestica* L. (European plum);
C: *P. cerasifera* Ehrh. (cherry plum, myrobolan);
D: *P. spinosa* L. (blackthorn, sloe).

(A)

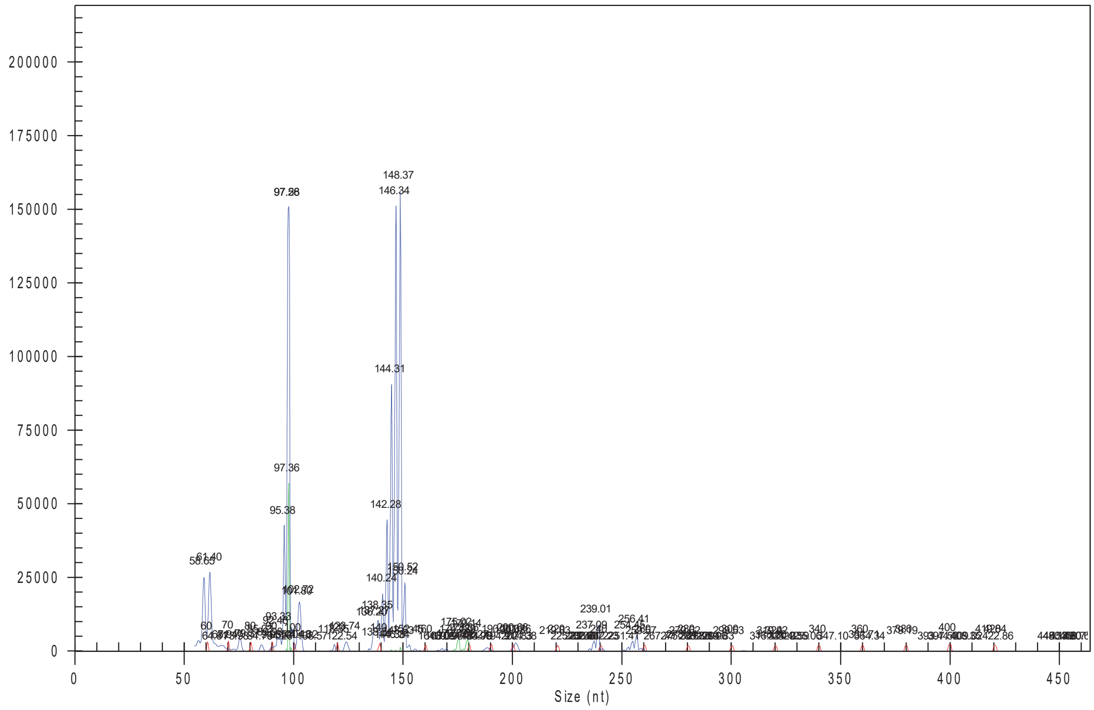

(B)

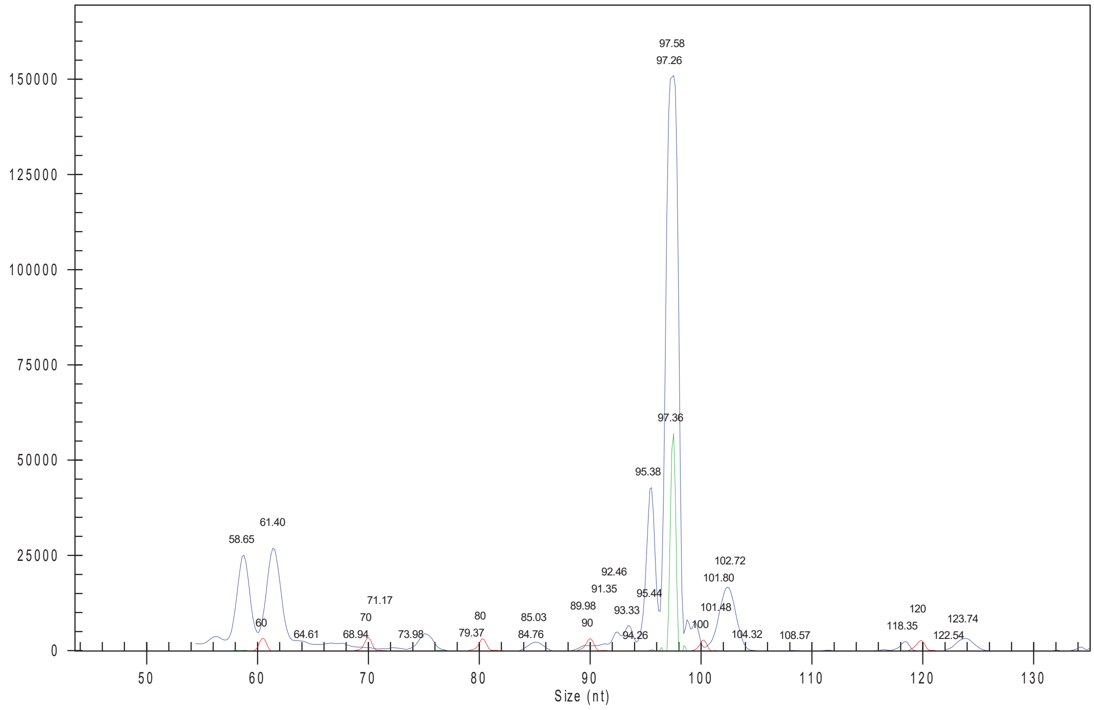

continued

(C)

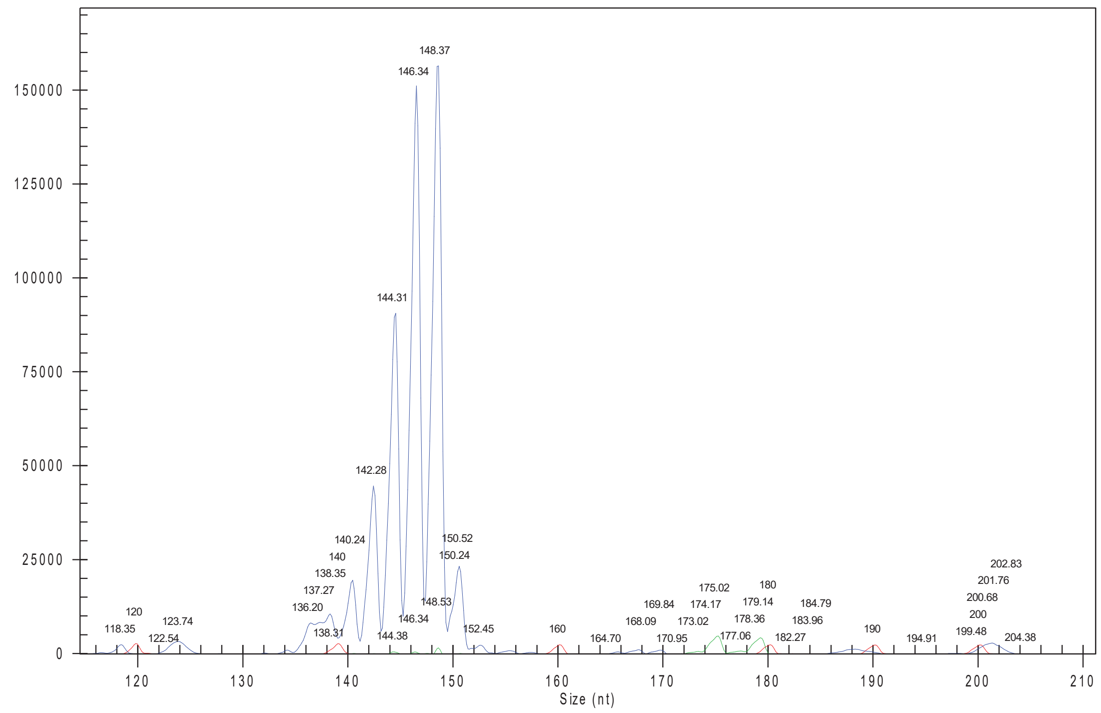

Figure S2: Example of two loci amplification: electropherogram of a diploid accession Plum 51 showing the amplification of UDP96-005 SSR (colored with dye blue), the primer pairs amplified two loci. A: general profile of the electropherogram; B: zoomed profile of the first locus; C: zoomed profile of the second locus. Observed alleles: 98, 146, 148 were coded as 98/98, and 146/148.

(A)

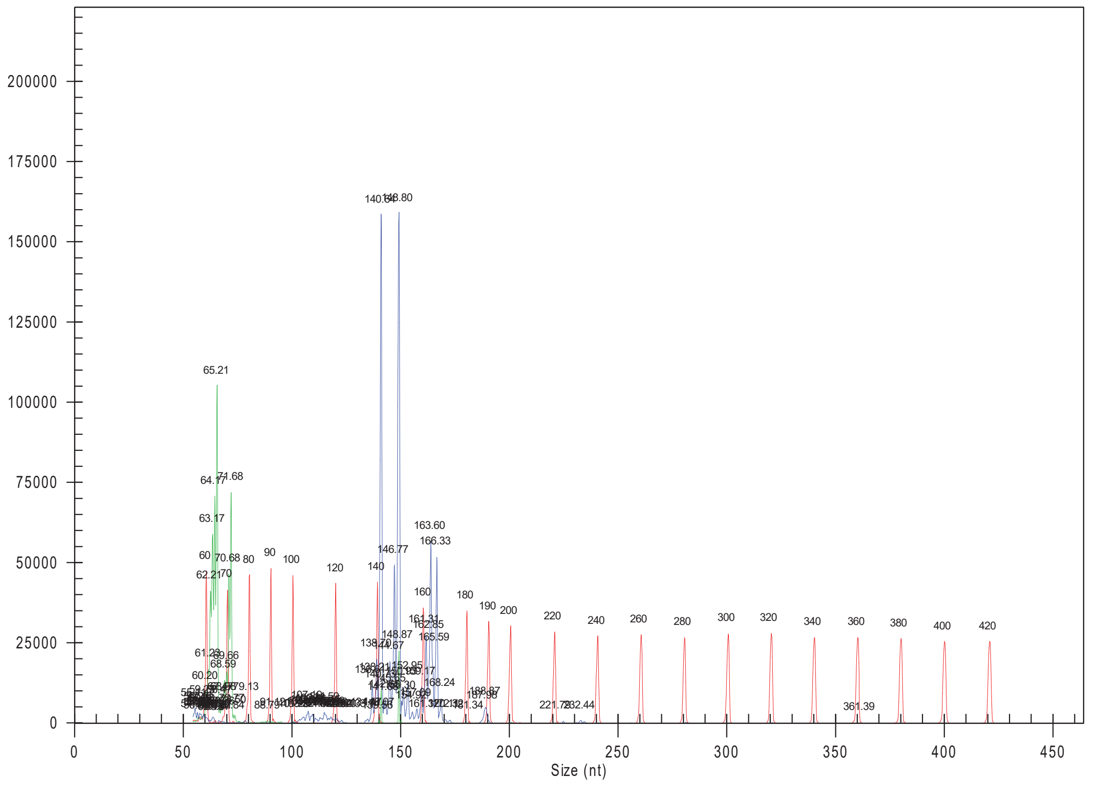

(B)

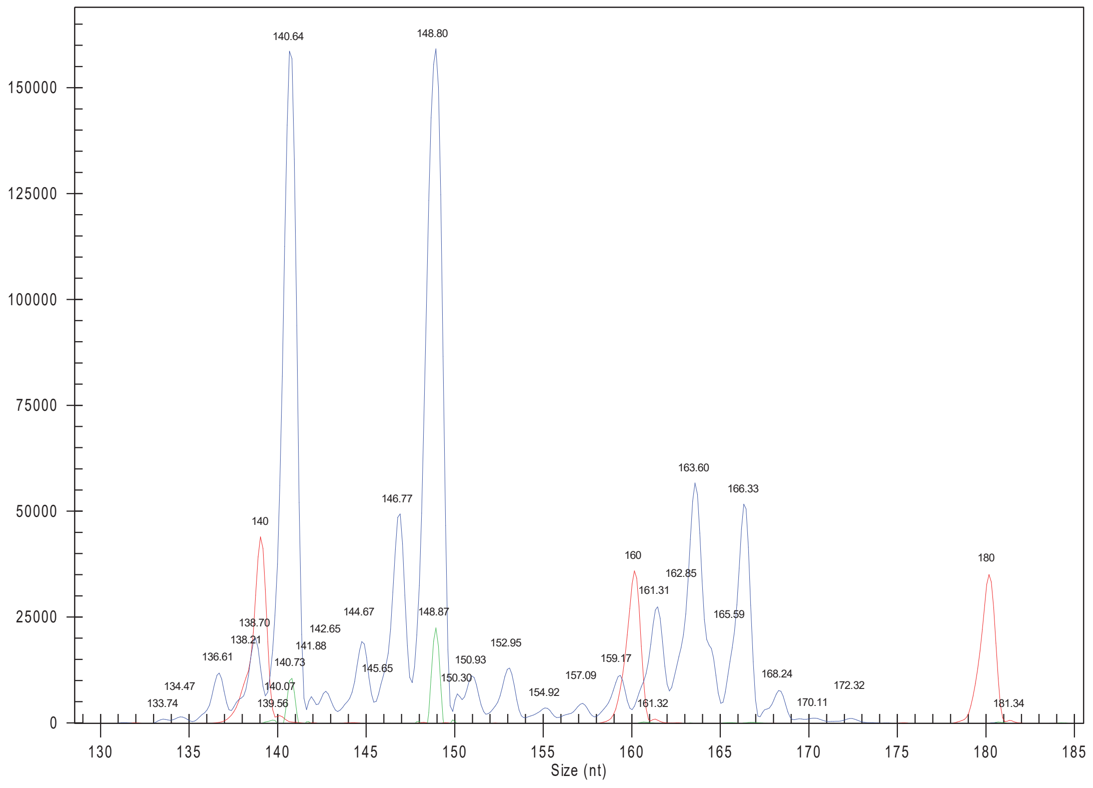

Figure S3: Example of allele dosage: electropherogram of an hexaploid accession ‘*Prunus insititia* Tersen’ (178) showing the amplification of UCD-CH17 SRR (colored with dye blue). A: general profile of the electropherogram; B: zoomed profile. Observed alleles: 141, 149, 163, 167 were coded as 141/141/149/149/163/167.

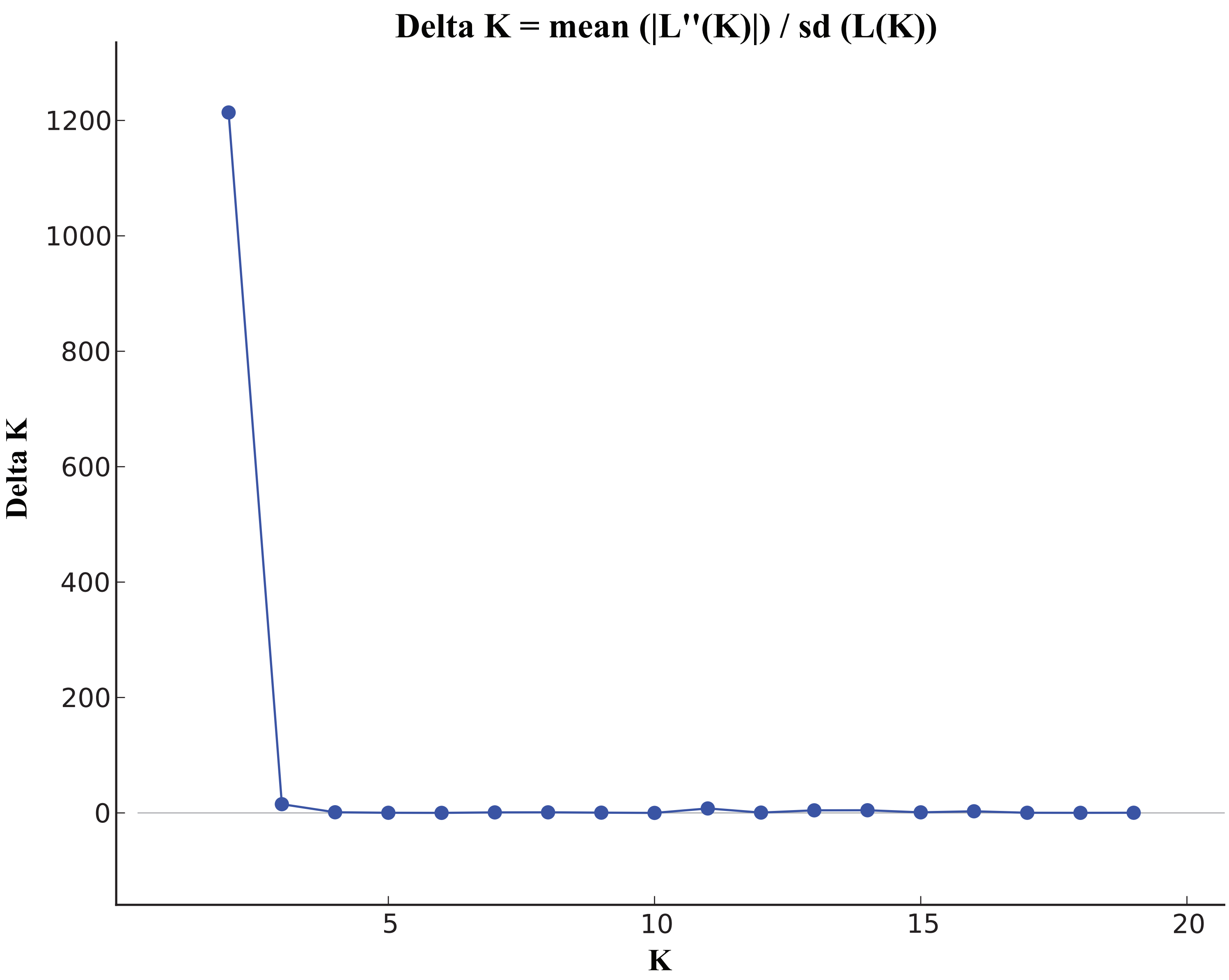

Figure S4: Graphical method, as in Evanno et al. (2005), allowing the detection of the number of groups K on the whole data set using ΔK .

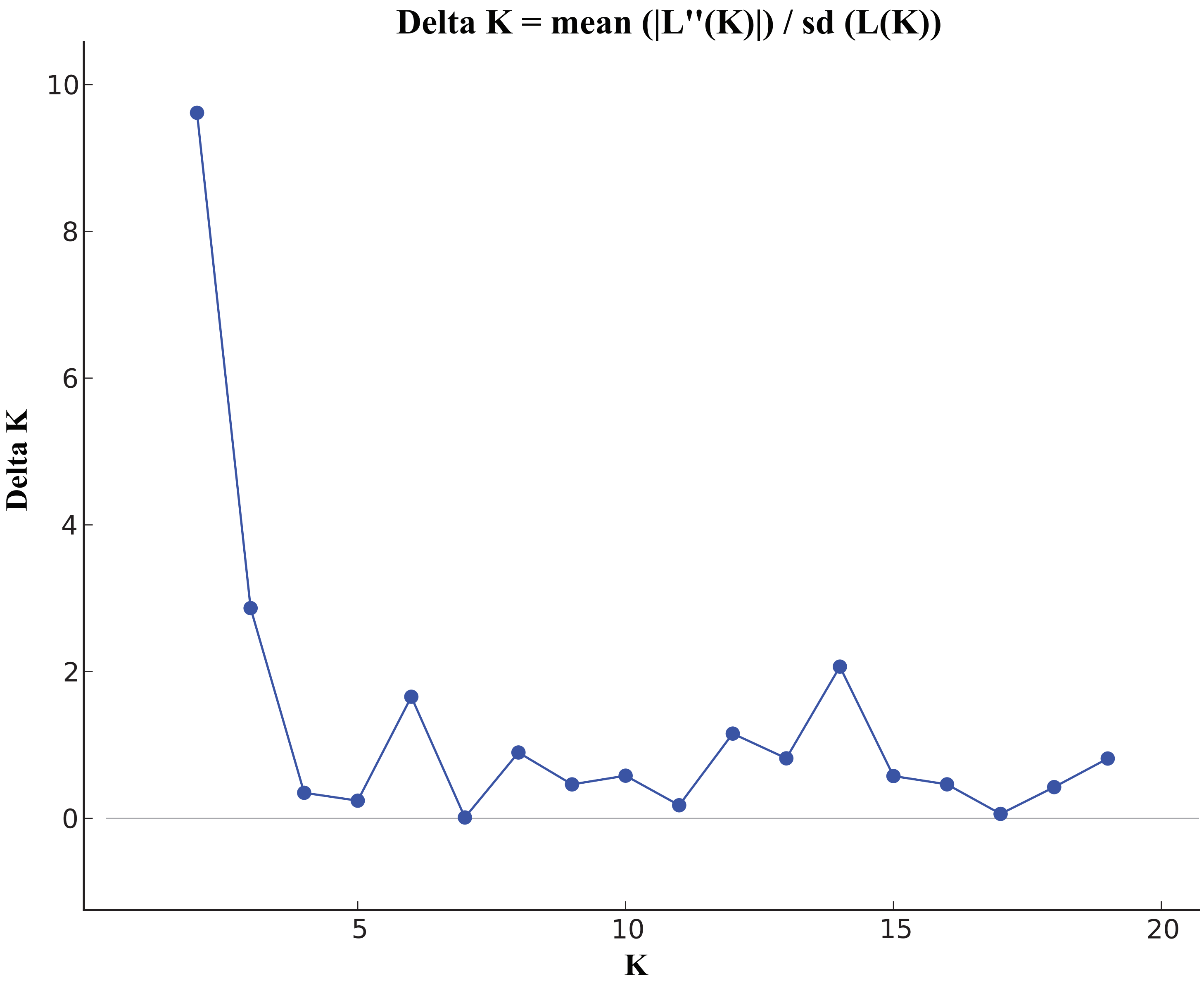

Figure S5: Graphical method, as in Evanno et al. (2005), allowing the detection of the number of groups K using ΔK on the *P. domestica* data set.

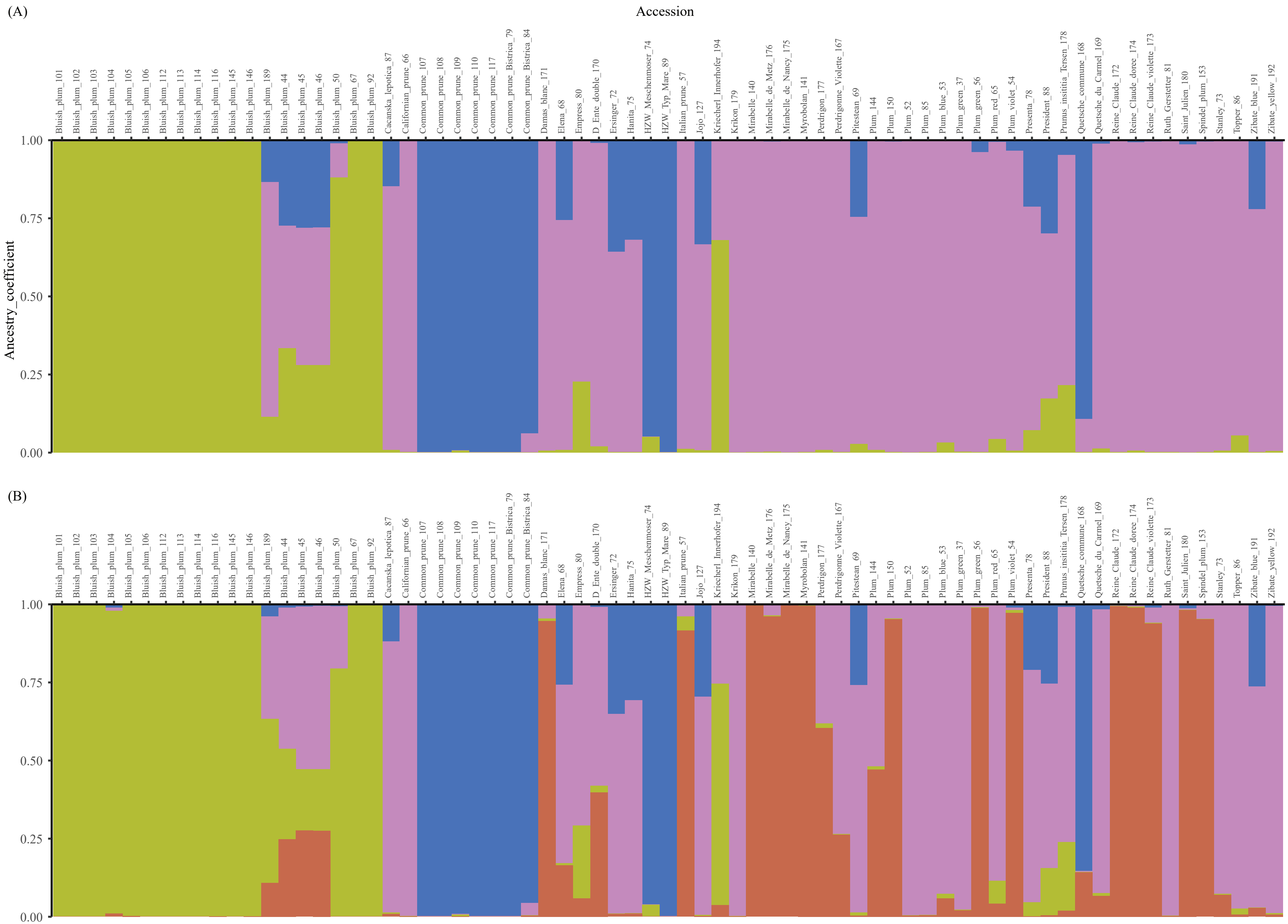
(A)
Accession
Ancestry_coefficient
1.00
0.75
0.50
0.25
0.00
Bluish_plum_101
Bluish_plum_102
Bluish_plum_103
Bluish_plum_104
Bluish_plum_105
Bluish_plum_106
Bluish_plum_112
Bluish_plum_113
Bluish_plum_114
Bluish_plum_116
Bluish_plum_145
Bluish_plum_146
Bluish_plum_189
Bluish_plum_44
Bluish_plum_45
Bluish_plum_46
Bluish_plum_50
Bluish_plum_67
Bluish_plum_92
Cacanska_lepotica_87
Californian_prune_66
Common_prune_107
Common_prune_108
Common_prune_109
Common_prune_110
Common_prune_117
Common_prune_Bistrica_79
Common_prune_Bistrica_84
Damas_blanc_171
Elena_68
Empress_80
D_Ente_double_170
Ersinger_72
Hanita_75
HZW_Meschenmoser_74
HZW_Typ_Mare_89
Italian_prune_57
Jojo_127
Kriecherl_Innerhofer_194
Krikon_179
Mirabelle_140
Mirabelle_de_Metz_176
Mirabelle_de_Nancy_175
Myrobolan_141
Perdrigon_177
Perdrigone_Violette_167
Pitestean_69
Plum_144
Plum_150
Plum_52
Plum_85
Plum_blue_53
Plum_green_37
Plum_green_56
Plum_red_65
Plum_violet_54
Presenta_78
President_88
Prunus_insititia_Tersen_178
Quetsche_commune_168
Quetsche_du_Carmel_169
Reine_Claude_172
Reine_Claude_doree_174
Reine_Claude_violette_173
Ruth_Gerstetter_81
Saint_Julien_180
Spindel_plum_153
Stanley_73
Topper_86
Zibate_blue_191
Zibate_yellow_192
(B)

continued

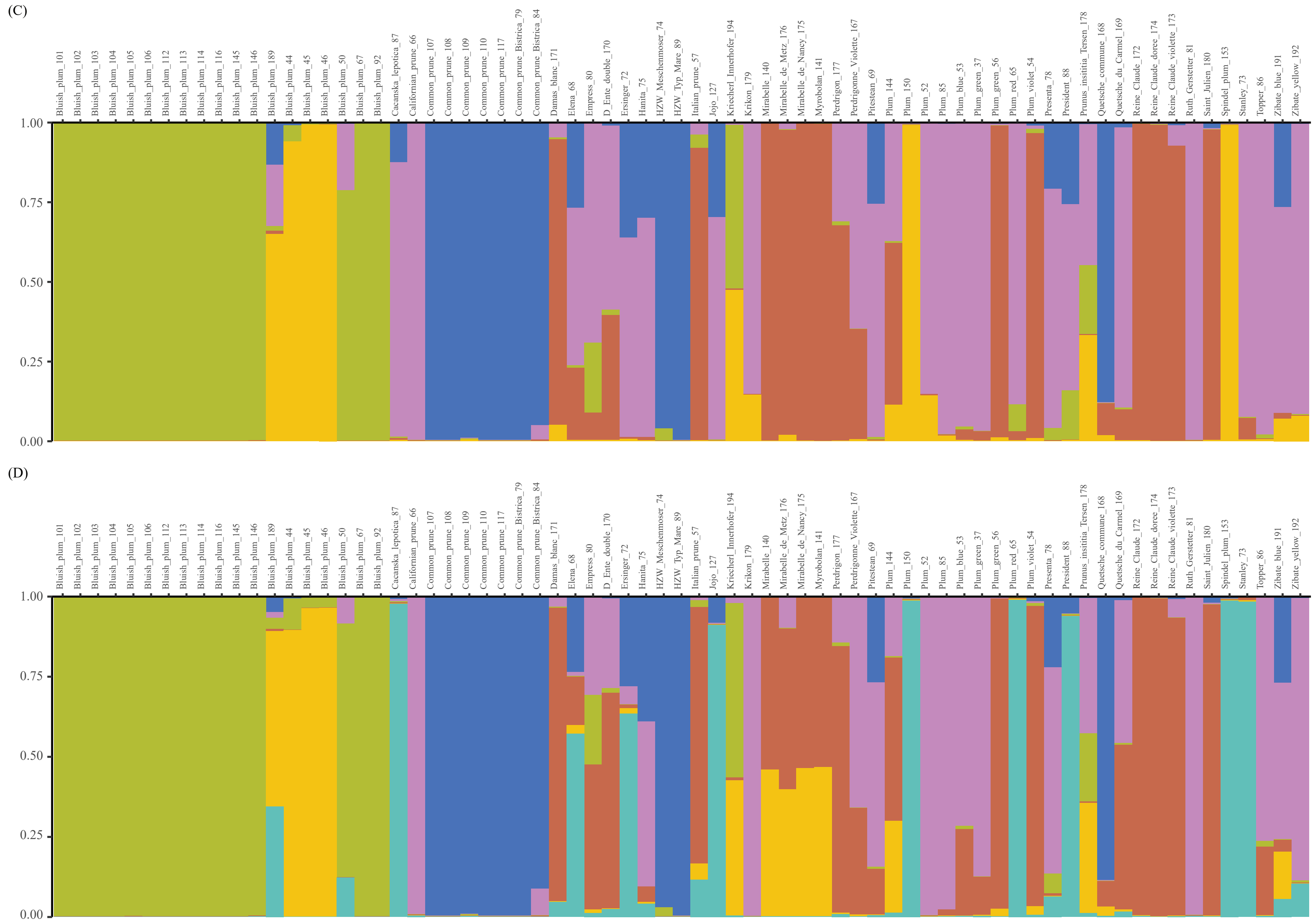

Figure S6: Graphical display of the results of the Structure analyses on the *P. domestica* data set. Proportions of ancestry of accessions for: K =3 (A); K=4 (B); K=5 (C) and K=6 (D). Each genotype is represented by a vertical bar. The groups are depicted by different colors.

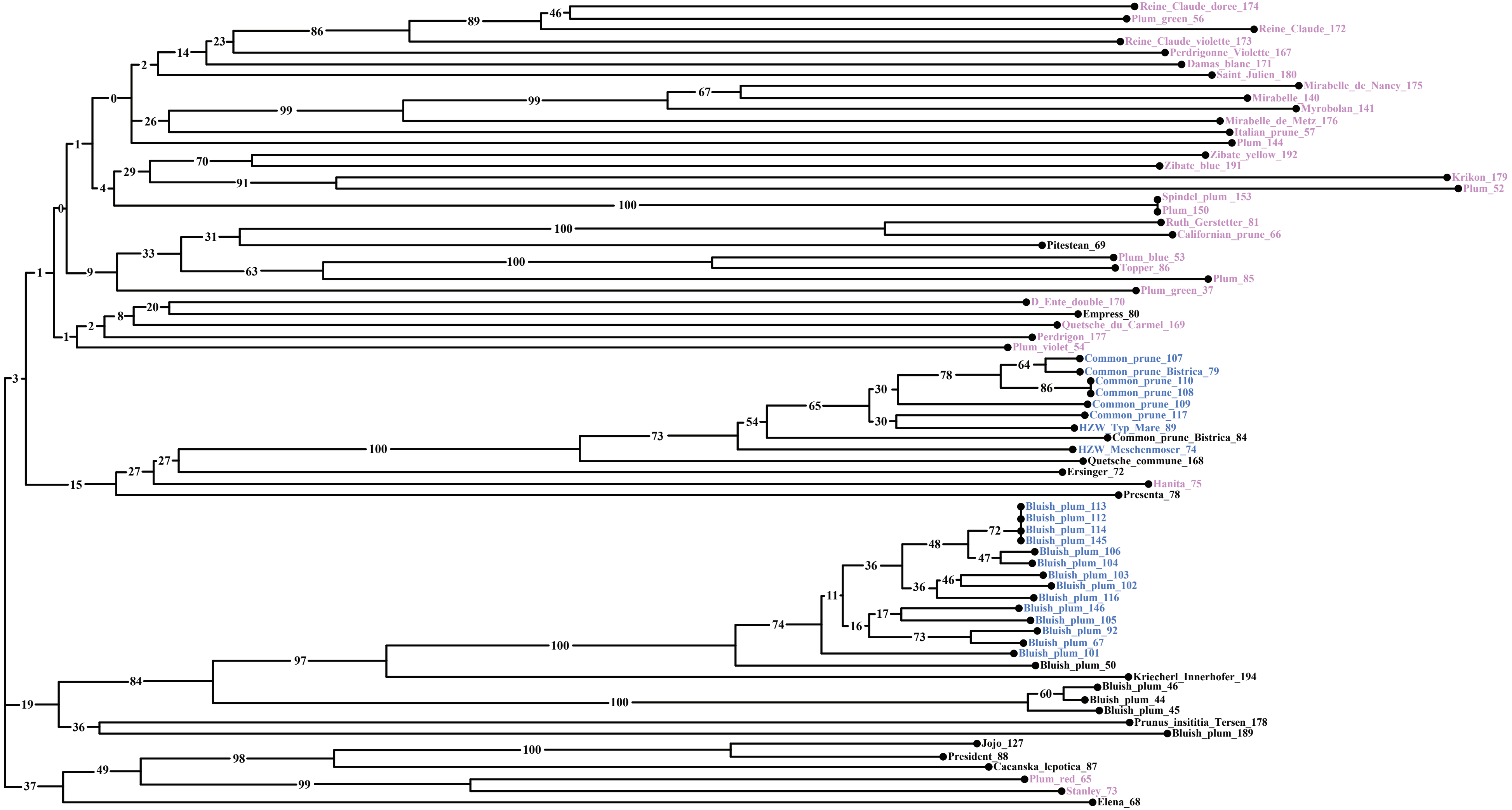

Figure S7: Neighbor-Joining tree based on the dissimilarity matrix calculated from the data set of 11 SSRs for 71 *P. domestica* accessions using the Sokal and Michener index. The colors of the accessions indicated the clusters obtained by Bayesian analysis for K=2: Bluish plum accessions and common prunes collected in Slovenia (blue), plums genetically very diverse (violet), accessions in black correspond to the admixed accessions.
